# Microalgae Biofuel for a Heavy-Duty Transport Sector within Planetary Boundaries

**DOI:** 10.1021/acssuschemeng.3c00750

**Published:** 2023-06-13

**Authors:** Richard Cabrera-Jiménez, Victor Tulus, Jordi Gavaldà, Laureano Jiménez, Gonzalo Guillén-Gosálbez, Carlos Pozo

**Affiliations:** † Departament d’Enginyeria Química, Universitat Rovira i Virgili, Av. Països Catalans 26, 43007 Tarragona, Spain; ‡ Institute for Chemical and Bioengineering, Department of Chemistry and Applied Biosciences, ETH Zürich, Vladimir-Prelog-Weg 1, 8093 Zürich, Switzerland

**Keywords:** microalgae, biofuels, LCA, planetary boundaries, renewables, biosphere integrity, human health

## Abstract

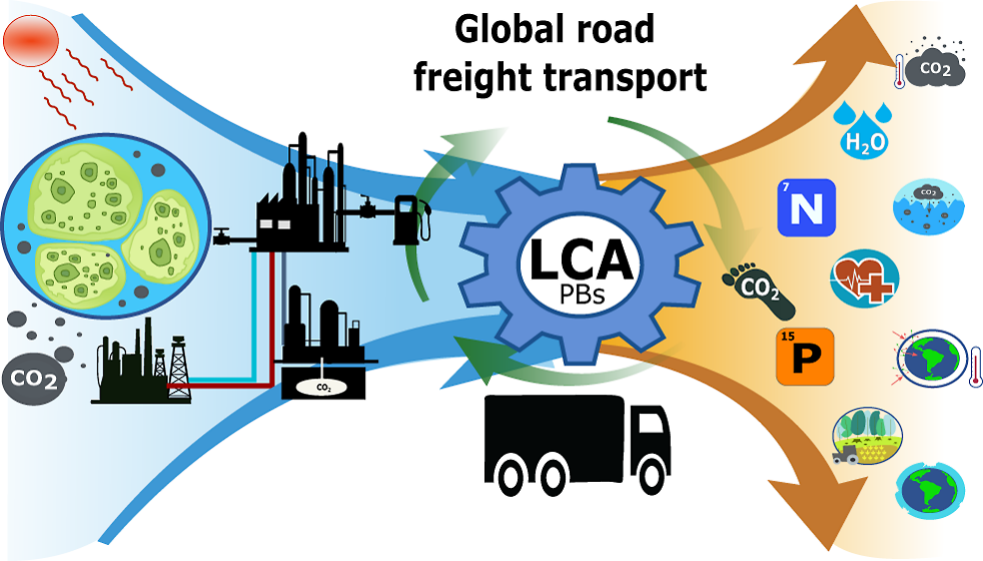

In this contribution, we study the extent to which 68 scenarios for microalgae biofuels could help the heavy-duty transport sector operate within planetary boundaries. The proposed scenarios are built considering a range of alternative configurations based on three types of fuel production processes (i.e., transesterification, hydrodeoxygenation, and hydrothermal liquefaction), different carbon sources (such as natural gas power plants and direct air capture), byproduct treatments, and two electricity mixes. Our results reveal that microalgae biofuels could significantly reduce the environmental and human health impacts of the business-as-usual (fossil-based) heavy-duty transport sector. Moreover, relative to standard biofuels that show large land-use requirements, we find that microalgae biofuels also decrease the damage on biosphere integrity substantially. Notably, pathways resorting to hydrodeoxygenation of microalgae oil and direct air capture and carbon storage could reduce the current impact induced globally on climate change by the heavy transport by 77%, while attaining six-fold reductions in biosphere integrity impacts, both relative to conventional biofuels.

## Abstract 2

We explore 68 microalgae-based biofuel production routes with the aim of identifying sustainable options for the freight transport sector.

## 1 Introduction

In a context where the transport sector is responsible for 22% of the global carbon emissions generated,^1^ electric vehicles emerge as a promising alternative for sustainable mobility. While this alternative is suitable for low-range vehicles used for urban mobility,^2^ the current state-of-the-art of batteries^3^ limit fuel substitution in heavy-duty vehicles that tend to travel longer distances, making liquid fuels from bio-based feedstock promising candidates for reducing the environmental impact exerted by this sector.^4^

Currently, the world production of biofuels is based on agricultural crop biomass causing competition for the available land between fuel and food production.^5,6^ This exacerbates the risk of losing biodiversity and ecosystem services. Some of these problems could be avoided by producing liquid fuels from microalgae, which, compared to conventionally farmed biofuels, shows advantages such as rapid growth and low or marginal use of land. Carbon sequestration and the capacity of self-producing energy using byproducts are additional advantages.^7,8^

Microalgae are carbon-fixing microorganisms that require adequate CO_2_ concentrations to thrive, typically provided through CO_2_-enriched air flow with concentrations in the range of 0.8–10% volume.^9^ Considering that the atmosphere contains a low concentration of CO_2_ (i.e., around 0.041%), supply of atmospheric air would not be enough, and additional CO_2_ should be injected to prevent an insufficient concentration that would limit productivity. CO_2_ can be obtained by capture techniques, either used at point sources such as steam methane reforming, NH_3_ production, or natural gas power plants (NGP),^10^ or by using direct carbon capture from the air (DAC).^8^ In both cases, engaging microalgae production with CO_2_ capture has the potential to reduce the negative effect on climate change compared to the use of conventional fuels, either by avoiding some carbon emissions at point sources or by resorting to atmospheric (instead of fossil) CO_2_ when using DAC. Another interesting possibility is the recovery and storage of the carbon embedded into the microalgae byproduct, which otherwise would be released back into the atmosphere upon natural decomposition. This can be done by capturing the CO_2_ emitted during the use of the residual biomass for cogeneration, and storing it in a geological deposit,^10^ thus converting the biofuel production process into a carbon capture utilization and storage process.

Among the different biomass conversion routes, the two routes that stand out are (1) the production of biodiesel (BD) from a solvent-based lipid extraction with a subsequent transesterification and (2) the production of green (renewable) diesel.^11^ In turn, green diesel can be produced by two processes: hydrodeoxygenation after lipid extraction (HDO) or hydrothermal liquefaction (HTL). For the HTL process, the production of biofuel does not require a previous lipid extraction process, allowing the use of feedstocks with up to 20% moisture content avoiding the use of dehydration and drying pretreatments.

Biofuel production from microalgae is an energy-intensive process, where most impacts occur either upstream (e.g., energy production for water recirculation) or downstream (e.g., biofuel combustion in the vehicle engine) of the main facility. This calls for the application of life cycle assessment (LCA)^12^ as an essential tool to identify the most sustainable technologies for microalgae-based biofuel production.

Since its development, LCA has allowed for comprehensive environmental analyses of processes and products, covering activities from feedstock extraction to waste management. So far, several impact assessment methods have been put forward (e.g., CML and ReCiPe), yet they lack absolute thresholds to elucidate whether a given system should be deemed sustainable. To overcome this limitation, here we focus on an absolute environmental sustainability assessment (AESA) method, which bridges conventional LCA principles with the concept of Planetary boundaries (PBs), developed by Rockström et al.^13^ and Steffen et al.^14^ The PB framework aims to quantify the absolute environmental sustainability level of human activities by proposing nine bio-geophysical boundaries for the Earth system that define a “safe operating space for humanity” (SOS). These boundaries represent quantitative thresholds whose transgression could alter the current state of the Earth in an irreversible manner.^14^ So far, PBs have been established for climate change, change in biosphere integrity, stratospheric ozone depletion, ocean acidification, biogeochemical flows, land-system change, freshwater use, atmospheric aerosol loading, and the introduction of novel entities. The transgression of the SOS undermines the resilience of ecosystems that support human well-being and has repercussions on human health problems at different scales.^14^

In the context of biofuels, several studies applied LCA to evaluate different production technologies. Most of them use a cradle-to-tank scope, whereby the focus is on the production of biofuels through transesterification processes, including also upstream activities (e.g., biomass production), but excluding the combustion stage.^12,13^ Some of these studies focus only on particular processes, such as microalgae production,^15^ microalgae oil production,^16^ or fuel production,^17^ where the carbon source, and its downstream repercussions are mostly ignored. The LCA scope was extended in the contributions by Batan et al.^18^ and Ou et al.,^19^ applying a cradle-to-wheel approach. However, these studies focused only on greenhouse gas (GHG) emissions, thereby neglecting other relevant impacts such as land or water use. Given that electricity consumption and CO_2_ supply are among the most operationally and economically significant factors in microalgae biofuel production, we include them in the present work. Yue et al.^20^ and Bennion et al.^17^ already explored these factors, but unlike them, we look into the use of renewables for electricity generation, which could bring additional environmental advantages.

For the first time, this contribution studies the transformation of microalgae into fuels through the lens of the PBs. Notably, we consider three different types of fuels (BD, HDO, and HTL) under 68 scenarios combining different carbon sources (i.e., CO_2_ from a NGP and DAC), byproduct treatments (i.e., biomass combustion, as well as biogas production and combustion), and electricity mixes (i.e., 2020 global mix, electricity mix projected under the sustainable development for 2040), reaching a level of breadth in the analysis way above previous studies. We combine the principles of LCA and an AESA based on the PBs, adopting a cradle-to-wheel perspective. Our study goes beyond GHG emissions, embracing other impacts on key Earth-system processes while also covering impacts on human health. Overall, this work evaluates the potential of microalgae biofuels to reduce impacts on the environmental and human health compared to both fossil fuels and conventional biofuels, with particular attention to impacts on Earth-system processes that are currently transgressed by anthropogenic activities (i.e., climate change, biosphere integrity, land-system change, and biogeochemical flows).

## 2 Methodology

The present study focusses on the AESA of the production of biofuels from microalgae and their use in the heavy-duty transport, always considering a cradle-to-wheel perspective (i.e., thus including the combustion phase).

**Figure 1:**
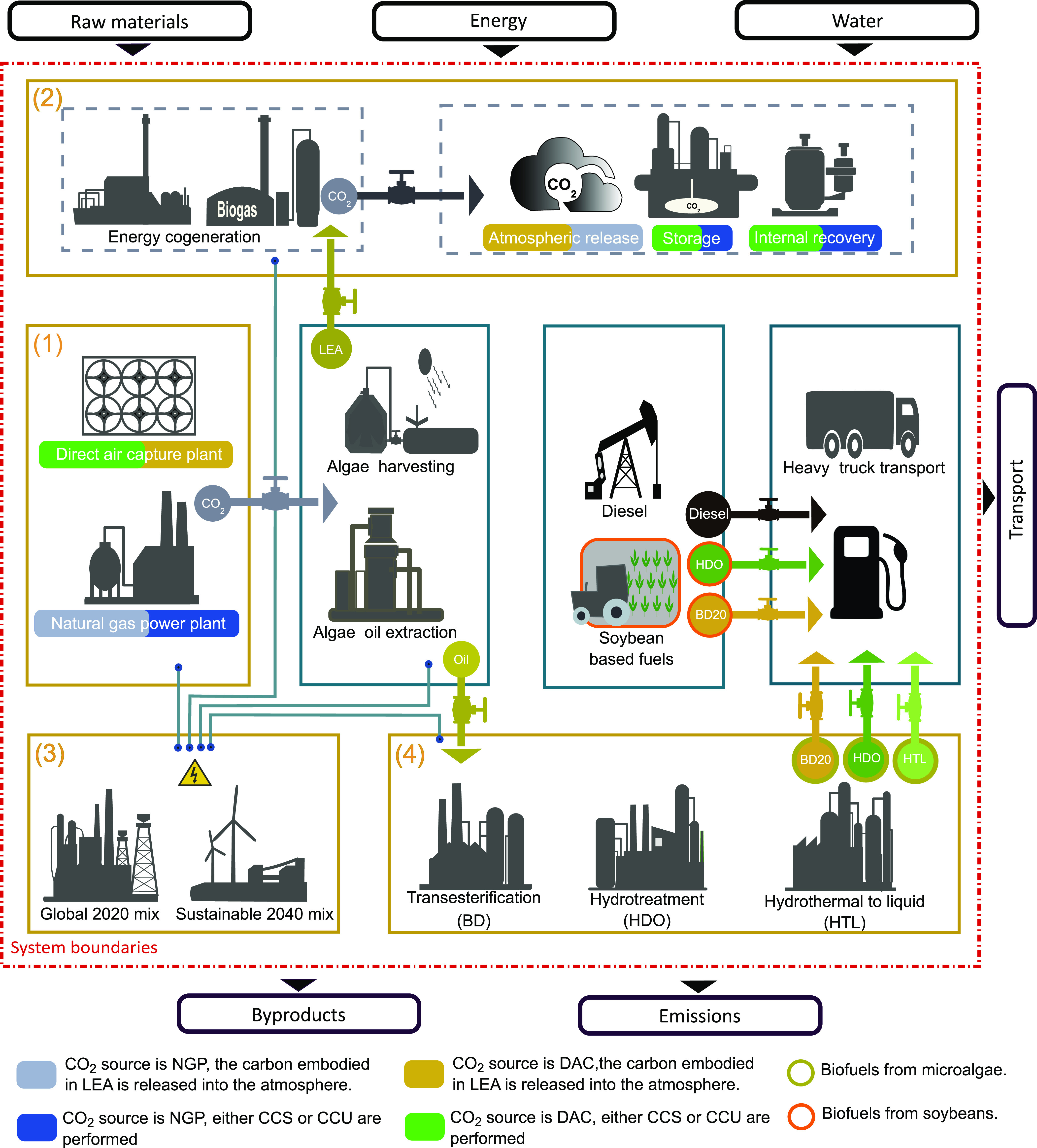
Conceptual framework for biofuel production from microalgae considering 68 scenarios based on four technological decisions: (1) carbon feedstock from carbon captured from DAC and a power plant; (2) carbon storage, carbon utilization, and carbon emissions considering the end of life CO_2_ from lipid-extracted algae (LEA); (3) 2020 global electricity mix and sustainable 2040 electricity mix; and (4) transesterification, hydrotreatment, and hydrothermal to liquid as biofuel conversion processes.

As shown in Figure 1, the 68 transformation scenarios of microalgae to biofuels are based on different combinations of alternatives for four technological decisions related to: (i) the source of CO_2_, (ii) the use of byproducts such as lipid-extracted algae (LEA), CO_2_, gasoline, electricity, or thermal energy, (iii) the electricity mix, and (iv) the type of fuel produced.

The first technological decisions affect the selection of a source for the CO_2_ that will be supplied to the microalgae to satisfy the carbon requirements and ensure that microalgae will never be CO_2_-limited throughout the course of cultivation. This can be obtained either from DAC or from NGP. The literature suggests that lower CO_2_ concentrations result in higher lipid production.^21,22^ Despite this, we consider compression and pumping of pure CO_2_ for consistency with the approach followed in the GREET database, from where we source most of our data.

The second technological decision concerns the potential utilization of the byproduct generated during the extraction phase of BD and HDO process, the so-called LEA. Three main alternatives are considered for LEA: (i) combustion to partially supply heat and electricity for self-consumption; (ii) production of methane using an anaerobic digester with subsequent biogas combustion; and (iii) disregarding the utilization of LEA. For this last option, we assume that the CO_2_ embedded in the LEA would be released as biogenic carbon back into the atmosphere as a result of natural decomposition. Conversely, the first two options generate CO_2_ as a byproduct during LEA or biogas combustion, which can be exploited in three different ways: (i) capture and utilization (CCU) in the cultivation process as a source of CO_2_, (ii) capture and storage in geological reservoirs (CCS), and (iii) direct emission to the atmosphere. Overall, this translates into seven practical alternative pathways for byproducts in BD and HDO routes. In the case of the HTL scenario, the data source considers a wood-microalgae mixture^23^ as feedstock. In this case, CO_2_ is the only byproduct generated, for which we consider the same three pathways as for the CO_2_ stemming from LEA or biogas combustion, i.e., CCU, CCS, or direct emission (i.e., no capture).

The third technological decision is related to the electricity mix that will supply the electricity requirements of the processes explicitly modeled in the study (i.e., foreground processes only). In this regard, two scenarios are considered: the 2020 global electricity mix (M2020) and sustainable global mix for 2040 (M2040).^24^ This technological decision is of utmost importance not only because the production of algae oil fuel is intensive in the electricity consumption (6.37 MJ/L of HDO; compared to 0.29 MJ/L of HDO for soybean-based fuel) but also because the decarbonization of the electricity mix might potentially decrease impacts on the control variables of the PBs.^25^

Finally, the fourth technological decision is devoted to the transformation of microalgae into a particular type of biofuel. Options considered include (i) biodiesel production from a solvent-based lipid extraction, with a subsequent transesterification and posterior blending with diesel in a 20% v/v blend (BD20), (ii) renewable diesel production by hydrodeoxygenation after the lipid extraction, and (iii) renewable diesel based on HTL of the microalgae.

Different alternatives for cultivation, drying, and extraction technologies were also considered at an earlier stage of the study (see Table S31) but were disregarded due to their low technology readiness levels (TRLs); therefore, they are not considered in the aforementioned scenarios. The alternatives selected for these processes correspond to the technologies with the highest TRL, namely, open pond technology for microalgae cultivation, centrifugation and flocculation for microalgae drying, and wet solvent extraction for the oil extraction process.

Figure 1 summarizes the 68 scenarios for microalgae-based biofuels combining different alternatives for CO_2_ sources (two); the electricity mix (two); and potential routes for the exploitation of LEA and/or CO_2_ byproducts (seven in the case of BD and HDO and three for HTL). We also consider three additional scenarios for the sake of comparison, consisting of diesel from fossil sources, which we label as the business-as-usual (BAU) scenario; and biofuels based on soybean (i.e., BD and HDO). The addition of these three scenarios to the microalgae scenarios adds up to 71 scenarios in total.

For the sake of readability, the different scenarios will be named after colors according to the origin of the CO_2_ supplied to the microalgae and the management of the CO_2_ byproduct as follows. GREY will be used when the CO_2_ source is NGP and the carbon embodied in LEA is released into the atmosphere, BLUE when the CO_2_ comes from NGP and either CCS or CCU is performed, YELLOW when CO_2_ comes from DAC and the carbon embodied in LEA is released into the atmosphere, and finally GREEN when CO_2_ comes from DAC and CCS or CCU is performed. These colors will be complemented with a superscript related to the electricity mix (i.e., M2020 or M2040) and a subscript associated with the management of the CO_2_ byproduct as follows: ACR when CO_2_ is released to the atmosphere after cogeneration of LEA, CCU when CO_2_ is reused in the cultivation process, and CCS when CO_2_ is stored in a geological reservoir. If CCU or CCS are not considered, or if there is no LEA cogeneration, we assume that the CO_2_ contained in the LEA is released into the atmosphere upon its natural decomposition, and assign NoCCU as subscript to the corresponding label. Finally, the color label will be accompanied by a suffix describing the type of the biofuel production process (i.e., HDO, BD, or HTL). Although two cogeneration scenarios are considered (i.e., LEA combustion and biogas production), to facilitate the discussion, only scenarios considering cogeneration by combustion are described in the main manuscript, leaving scenarios from biogas combustion in the Supporting Information.

We acknowledge that such a large number of scenarios could motivate the use of optimization-based approaches to identify the ones showing a Pareto optimal performance.^5^ However, we preferred to retain the exhaustive analysis of all the scenarios to elucidate also those achieving a similar performance to optimal cases. These scenarios, although suboptimal under the assumptions considered here, could become optimal under different conditions for plant location, season, or distances of CO_2_ sources and storage sites relative to the biofuel production facility.

### 2.1 LCA and Planetary Boundaries

LCA quantifies the environmental impacts of products, processes, and services over their entire life cycle, covering a wide range of potential damages. To apply it, we follow ISO 14040 and 14044 standards based on four steps for identifying environmental hotspots.^12,26^

The first LCA phase defines the goal and scope of the study. The goal of this environmental assessment is to quantify the absolute environmental sustainability level of the different scenarios for microalgae-based biofuel routes. To this end, we defined the annual world ton-km (tkm/yr) demand for road freight activities as the functional unit, considered equal to 35 trillion tkm/yr for 2022.^27^ We adopt a cradle-to-wheel scope, thus covering all activities upstream of fuel production, the production of fuels and byproducts, and the final use of the fuel in vehicles intended for road freight activities (i.e., long haul-heavy trucks, 17t). An economic allocation was used as the attributional method to allocate impacts among the different products generated by each activity.

The second phase of the LCA quantifies the main inputs and outputs (i.e., energy, raw material, byproducts, and emissions) crossing the system boundaries. Here, mass and energy balance information from previous studies were retrieved for activities in the foreground system, namely, carbon sequestration, microalgae cultivation, microalgae drying, byproduct recovery, fuel production, and fuel combustion. Data for cultivation and drying phases were obtained from previous studies,^23,28^ considering freshwater use for farming activities as culture media. Material and energy requirements for the oil extraction phase were obtained from the GREET database.^28^ This database provides harmonized values obtained from a microalgae production model that considers three types of algal strains: *Chlorella sorokiniana*, *Kirchneriella cornuta*, and *Scenedesmus obliquus*.^29^ Similarly, mass and energy requirements for CCS processes were modeled according to previous studies.^30−33^ Then, this information was combined with the corresponding background activities data from ecoinvent v3.7.1,^34^ using SimaPro v11,^35^ to calculate the life cycle inventories (LCIs) of the different scenarios modeled. Additional details on the modeling of these scenarios are provided in Section 2 of the Supporting Information.

The third phase of the LCA involves assessing the damage produced by the LCIs in different environmental categories. To this end, nine control variables referring to seven Earth-system processes were considered.^13^ Aerosol loading and novel entities were omitted as these Earth-system processes PBs are not yet quantified.^14^ Hence, considering a set *B* of nine control variables of the PBs and a set *S* of 71 scenarios, the environmental impact caused by each scenario *s* ∈ *S* in each control variable *b* ∈ *B* (IMP_*b*,*s*_) was calculated according to eq 1.
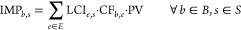
1Here, LCI_*e*,*s*_ represents the elementary flow *e* linked to the transportation of 1 ton of load across 1 km of distance through the use of the heavy-duty truck in scenario *s*. Elementary flows are referred to exchanges between the biosphere and the technosphere (e.g., kilograms of CO_2_ fossil emitted). Characterization factors CF_*b*,*e*_, computing the impact caused on control variable *b* by elementary flow *e*, were taken from Ryberg et al.^36^ for all control variables of PBs except for biosphere integrity. For biosphere integrity, we used the characterization factors developed by Galán-Martín et al.,^37^ considering two main stressors of biodiversity loss, i.e., direct land use and CO_2_ emissions. Finally, the product between LCIs and CFs is, in turn, multiplied by the global demand for road freight activities estimated for 2022 (PV, in tkm/yr) to determine the total impact linked to the functional unit.

Note that while PBs are defined at the planet level, variable EB_*b*,*s*_ considers only the impacts from a particular economic sector (i.e., heavy-duty transport sector). To harmonize this difference in scope, different downscaling approaches (e.g., egalitarian, utilitarian, acquired rights, or prioritarian)^38^ can be used to assign a share of the whole SOS to the specific system under study.^39^ However, sharing principles remain controversial, and there is no universal agreement on which or how they should be applied in practice.

Here, instead of using downscaling methods, we follow previous studies and simulate the global anthropogenic impact of the whole economy (IMP^GLO^) that would result from replacing the BAU scenario of the heavy-duty transport sector (IMPT^BAU^) by an alternative biofuel scenario (IMPT^ALT^).^37,40^ This is done by departing from the total current anthropogenic impact of the whole economy (EB^CUR^), subtracting from it the contribution of the sector under study in the BAU scenario, and then adding the impact of the alternative scenario for the same sector (eq 2).
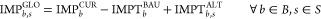
2Here, IMP_*b*,*s*_^GLO^ is the global impact of the whole economy in control variable *b* under scenario *s*; IMP_*b*_^CUR^ corresponds to the current anthropogenic impact level in control variable *b* (after subtracting the natural background level, as shown in Table S2); IMP_*b*_^BAU^ is the impact of the BAU scenario in control variable *b*; and IMPT_*b*,*s*_^ALT^ is the impact of scenario *s* in control variable *b*.

We stress that, in absolute sustainability studies, results are normalized relative to the maximum allowable impact to explicitly address the question of whether a system is environmentally sustainable in absolute terms in a given Earth-system process. Hence, with the global impact achieved in each scenario *s* at hand, we then calculate the PB footprint (PBF_*s*_) by comparing the global (predicted) anthropogenic environmental impact (IMP_*b*,*s*_^GLO^) with the SOS_b_ as defined by Steffen et al.,^14^ using eqs 3 and 4.
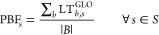
3
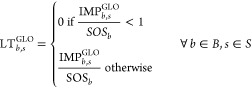
4Here, the global level of transgression (LT_*b*,*s*_^GLO^) shows the fraction of the SOS occupied by the whole economy for control variable *b* under scenarios *s* for the global transport sector. PBs not transgressed after sector substitution will show an LT_*b*,*s*_^GLO^ value of 0. To determine the level of transgression caused only by the transport sector (LT_*b*,*s*_^TRA^), IMPT_*b*,*s*_ will be used instead of IMP_*b*,*s*_^GLO^ in eq 4.

Finally, in step four of the LCA methodology, results are interpreted, and recommendations are made. In this case, we analyzed the LTs of the scenarios to identify the main hotspots, comparing their absolute environmental sustainability performance and determining whether they are truly sustainable. Section 3 of this manuscript is dedicated to this LCA phase.

### 2.2 Human Health Impacts

Although it is recognized that human health depends on safeguarding the natural systems that support human well-being,^41^ the PB framework omits impacts affecting human health directly. For this reason, as done in previous studies,^42^ we complement our study by including the “Human health” (HH) impact category from the ReCiPe 2016 method.^43^ In particular, we use the hierarchic perspective, which integrates impacts over a 100 year time horizon, thus quantifying the HH endpoint indicator in terms of Disability-Adjusted Life Years (DALYs).^43,44^ This endpoint indicator considers the following midpoint metrics: global warming, ozone formation, stratospheric ozone depletion, fine particulate matter, water consumption, ionizing radiation, human non-carcinogenic toxicity, and human carcinogenic toxicity.

### 2.3 Carbon Footprint and CO_2_ Balance

In addition to the PBs, we include in the analysis the well-known carbon footprint (CFP) indicator, which measures the total GHG emitted by a product over its life cycle, expressed in Gt of CO_2_ equivalent. Specifically, we follow the ReCiPe 2016 methodology for a time horizon of 100 years with the aim of comparing the results obtained from the ReCiPe methodology with the results from the corresponding control variable for the PB framework (i.e., atmospheric CO_2_ concentration and Earth’s energy imbalance).

In order to calculate the CO_2_-based PB control variables and the CFP, the benefits of CO_2_ uptake during biomass growth need to be properly assessed. This is a controversial issue^45^ since this uptake could be deemed as negative emissions depending on the system boundaries.

**Figure 2:**
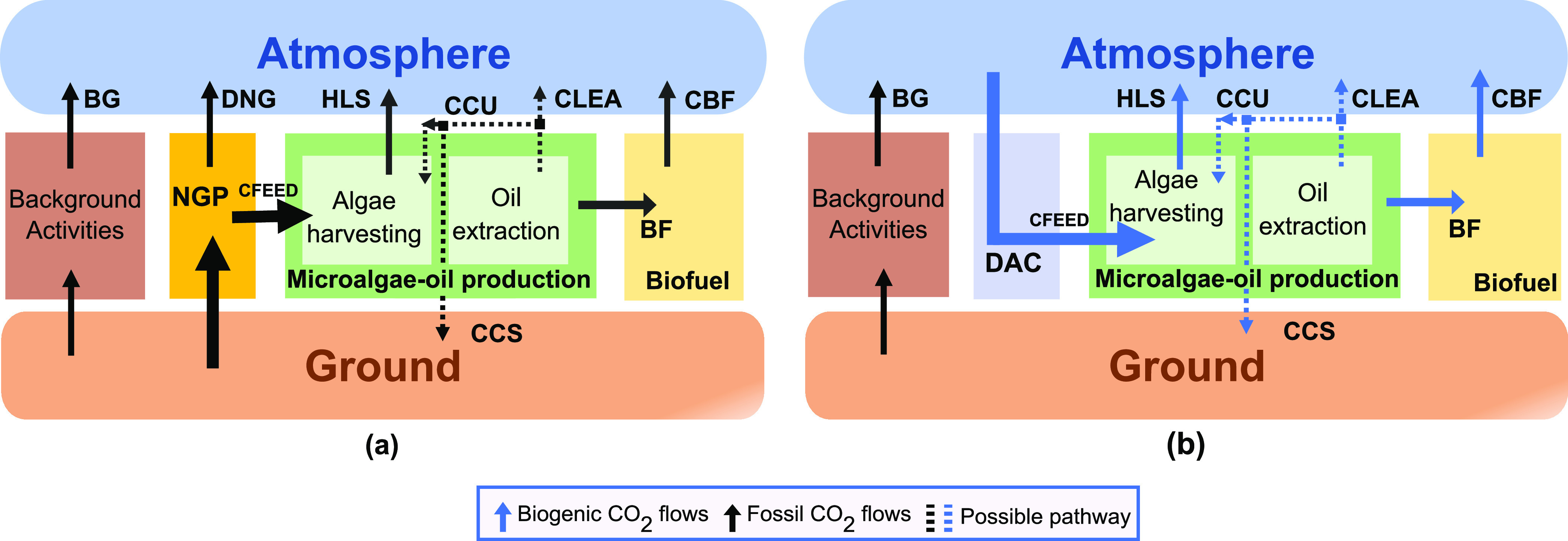
Carbon dioxide balance approach: (a) CO_2_ captured from fossil point sources, (b) CO_2_ captured directly from the air. NGP: CO_2_ flow from the natural gas power plant; DAC: CO_2_ flow from the direct air capture system; CCU: CO_2_ from carbon capture and utilization from LEA combustion; CLEA: CO_2_ flow linked to the emissions from the combustion of lipid-extracted algae residue; DNG: direct CO_2_ emissions from NGP; CFEED: CO_2_ flows for microalgae growth; HLS: CO_2_ losses from algae cultivation; BG: CO_2_ flow as emissions from background activities supplying materials and energy; CCS: CO_2_ flow for storage; BF: CO_2_ embodied into biofuel; and CBF: CO_2_ flow as emissions from biofuel combustion.

To perform a CO_2_ balance in the atmosphere, we first classify CO_2_ flows depending on the carbon source: (a) CO_2_ captured from fossil point sources (NGP) and (b) CO_2_ captured from direct air (DAC), as shown in Figure 2. These CO_2_ flows are initially used for microalgae growth (CFEED), where part of this carbon flow will be released into the atmosphere owing to leakages in open ponds (HLS), while the rest will be incorporated into algae biomass. In turn, part of this carbon will remain embodied in the final biofuel until it is burned (CBF), while the rest will be part of the so-called LEA byproduct.

In our system, LEA can be burnt to produce electricity and thermal energy for self-consumption. During this combustion, its carbon content will be released again as CO_2_, which can be partly captured and stored in a geological reservoir (CCS). For direct combustion of LEA, around 82% of the CO_2_ embodied is recovered, while in the case of biogas combustion, only a 33% can be retained. The remaining CO_2_, which is emitted to the atmosphere, is labeled as CLEA in the figure. Alternatively to the CCS scenario, we studied the option of reintroducing the CO_2_ captured during LEA combustion into the cultivation process (CCU), thus decreasing the initial required flow of CFEED.

Finally, the CO_2_ emissions coming from the background activities directly related to the system explicitly modeled (e.g., electricity for water recirculation in the open ponds) are labeled as BG.

Overall, the expressions used to perform the CO_2_ balance over the atmosphere are
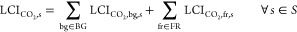
5
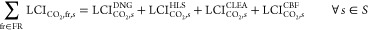
6where  denotes the CO_2_ flows from the background activities,  represents the CO_2_ flows from the foreground activities related to biofuel production,  is the CO_2_ embodied in the biofuel (stemming from the microalgae),  is the direct CO_2_ emissions incurred during farming, and  is the carbon embodied in the LEA. The latter would be released back into the atmosphere, either as a result of natural decomposition, or by scaping the capture system located downstream the LEA combustion or the HTL processes. Conversely, the carbon captured after LEA combustion or the HTL process can be stored as CO_2_ in geological reservoirs through CCS, thus decreasing the CLEA flowrate. The direct CO_2_ emissions from NGP () presented in Figure 2 are properly allocated between electricity and CO_2_ based on economic allocation (see Table S15 in the Supporting Information). In the case of using CO_2_ from DAC (Figure 2b), since the carbon used to grow the microalgae already comes from the atmosphere and no additional fossil carbon is required,  will not be accounted for. Note that the net CO_2_ in the atmosphere () will remain positive in both cases, since contributions from the background processes () will offset negative emissions in the foreground ().

## 3 Results and Discussion

The following section summarizes the results obtained for the different scenarios, that is, the impacts incurred by the different pathways of microalgae-based biofuels on different environmental categories (the PBs, the PBF, the CFP, and HH). For benchmarking purposes, we also show the results of the BAU and two conventional biofuel alternatives for heavy-duty transport, namely, conventional diesel and biofuels based on soybean oil.

### 3.1 Relative Contribution to the Safe Operating Space

**Figure 3:**
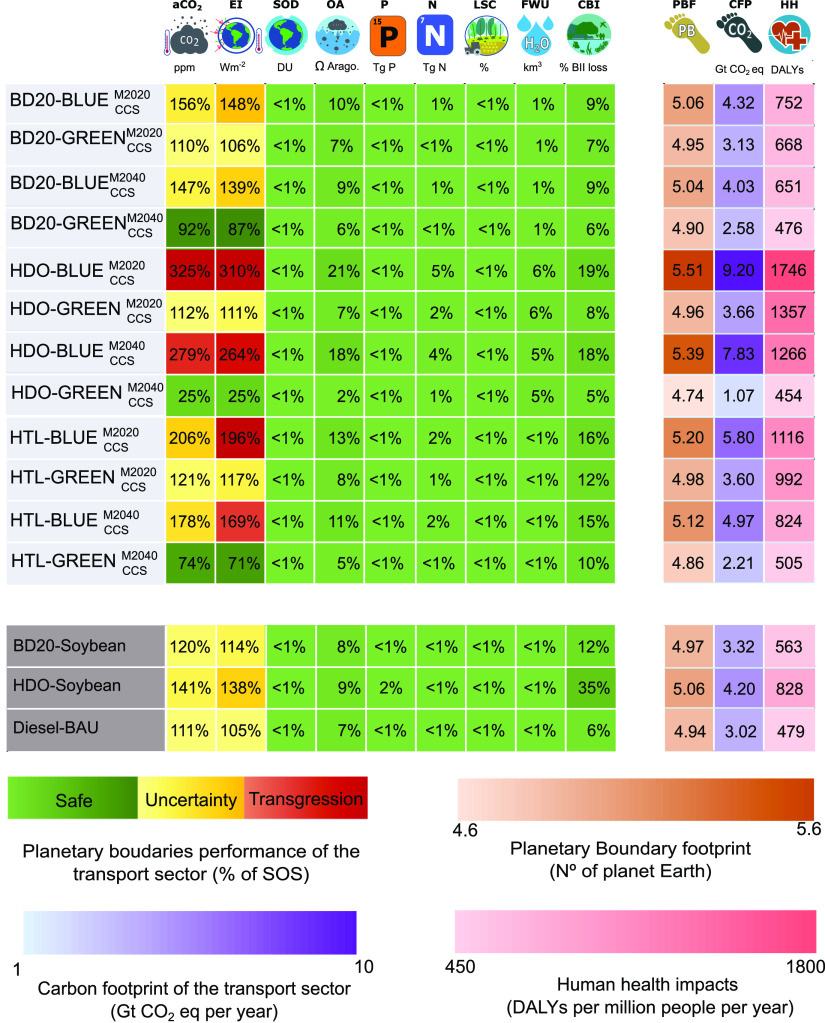
Impacts incurred by different scenarios for the global heavy-duty transport sector. Each row in the heatmap corresponds to a different scenario, as described by the labels in the first column: the fuel type (technological decision 4 in Figure 1) and the alternative selected for technological decisions 1, 2, and 3, respectively (e.g., CO_2_ source, electricity mix, and byproduct pathway). The remaining columns correspond to different impact categories as follows. The columns in the leftmost heatmap provide the level of transgression of the global SOS occupied by the heavy-duty transport sector alone. Then, the three rightmost columns, which are slightly separated, provide (from left to right) the PBF, the CFP, and HH. Each set of metrics uses a different color key according to the four bar legends, where the corresponding units are also indicated. Acronyms for the scenario labels are as follows. HDO: renewable diesel 100% vol from HDO; M2020: 2020 global electricity mix; M2040: sustainable electricity mix for 2040; CCS: carbon capture and storage in a geological reservoir; blue: NGP and either CCS or CCU is performed; green: DAC and CCS or CCU is performed (aCO_2_: atmospheric CO_2_ concentration; EI: energy imbalance at the top of the atmosphere; SOD: stratospheric ozone depletion; OA: ocean acidification; P: biogeochemical phosphorus flow-global; N: biogeochemical nitrogen flow-global; LSC: land-system change-global; FWU: freshwater use, global; CBI: biosphere integrity; PBF: planetary boundary footprint; CFP: carbon footprint; and HH: human health).

Figure 3 summarizes the level of transgression that would result from replacing the current transport sector with each of the different alternatives, expressed as the percentage of the SOS occupied by the heavy-duty transport sector in the nine PB control variables addressed. This level of transgression (i.e., variable LT_*b*,*s*_^TRA^) is calculated considering the lower, i.e., more stringent, limit of the uncertainty zone proposed by Steffen for the PB of every control variable.^14^ In turn, the color indicates the level of transgression of the control variable, green when LT_*b*,*s*_^TRA^ is below 100%, yellow when LT_*b*,*s*_^TRA^ is larger than one but within the uncertainty region for control variable *b*, and red when LT_*b*,*s*_^TRA^ exceeds the relaxed PB proposed for control variable *b* (see Table S3 for current values of control variable, along with their proposed PB). In addition, the PBF, the CFP, and HH impacts are also provided for each scenario. Note that, due to space limitations, we only provide here the results for 12 representative microalgae biofuel scenarios. The remaining 56 scenarios for HDO, BD20, and HTL are available in Section S3.1 of the Supporting Information.

#### 3.1.1 Conventional Fuels and Their Impact on the Heavy-Duty Transport Sector

According to the results obtained, the present heavy-duty transport sector (i.e., fueled by diesel) transgresses by a factor of 1.11, the SOS for CO_2_ concentration. Results are not better for conventional soybean-based biofuels, which transgress the same boundary by a factor of 1.20 or 1.41, depending on whether soybean is used to produce BD or HDO. This suggests that the higher the percentage of biofuel in the final blend (20% v/v for BD20 vs 100% v/v for HDO), the greater the impact associated with CO_2_ emissions. Note that we drop the label 100% to describe HDO and HTL scenarios since none of these scenarios uses a different blend.

These results show that the current heavy-duty transport sector alone transgressed the SOS of climate change for the whole economy and that the scenarios where soybean is used as biofuel do not decrease the transgression but rather increase it even further. This is illustrated in the PBF metric, where the global transgression achieves values of 4.97 and 5.06 for BD20 and HDO, respectively; even higher than that for the BAU (diesel) scenario (4.94). This is due to the high impact on climate change during the production of soybean oil, and the low energy return on the investment ratio obtained compared to fossil fuels (i.e., 20:1 for diesel compared to 2:1 in biodiesel).^46^

Although the use of fuels based on soybean in the heavy-duty transport sector does not transgress the SOS for the remaining PBs (e.g., stratospheric ozone depletion or ocean acidification), their contribution to the change in biosphere integrity is relevant (i.e., 12% of the global SOS for BD20 and 35% for HDO), leaving little room for the remaining economic sectors. These results highlight the importance of finding alternatives that can reduce the impact on several PBs concurrently. With this spirit, scenarios for biofuels from microalgae will be discussed next.

#### 3.1.2 Technological Decisions on the Production of Biofuels from Microalgae

Considering the implications of the first technological decision, i.e., the carbon capture technology used for the CO_2_ feedstock, the advantage of using DAC stands out (i.e., comparing GREEN vs BLUE). Despite capturing a certain amount of CO_2_ with DAC requires 1.6 times more energy than doing it from a natural gas power plant owing to the lower concentration of CO_2_ in the air compared to point sources,^47^ DAC emits up to 2.9 times less fossil CO_2_ in the life cycle. This is because, under the assumptions adopted in this work, all the CO_2_ from NGP is modeled as fossil carbon and, therefore, it is a positive flow that increases the atmospheric CO_2_ concentration. Hence, DAC-based pathways show a substantial improvement in terms of PBF over NGP-based pathways (e.g., 65% CO_2_ emissions in the case of the HDO-BLUE_CCS_^M2020^ pathway compared to the HDO-GREEN_CCS_^M2020^ pathway). This difference between pathways increases, even more, when the electricity mix is decarbonized (M2020 vs M2040). In the HDO-BLUE_CCS_^M2040^ pathway, CO_2_ emissions are 91% higher than that in the HDO GREEN_CCS_^2040^ pathway. Note that, even in DAC-based scenarios, where atmospheric CO_2_ is captured and stored, indirect emissions incurred to meet the energy demand of this process offset these emissions, ultimately generating a positive net flow of CO_2_ into the atmosphere. More information is provided in Section 3 of the Supporting Information.

The second technological decision, related to the potential exploitation of LEA and the associated CO_2_ emissions, leads to a pattern in the PBF metric. Scenarios entailing biogas production and combustion have a higher PBF (up to 6%) than those where LEA is directly burned. This is due to several factors, such as the fugitive emissions incurred during methane production or the lower CO_2_ recovered for CCU or CCS at the end of the process (i.e., 44% lower than after direct combustion). The former aspect is particularly impactful because methane has a higher characterization factor (or global warming potential) than CO_2_ on climate change. In addition, the final digestate slurry from the biogas production process is usually used as fertilizer,^48^ which means that its embedded carbon will be finally released into the atmosphere^45^ through biological processes.^49^ Additional details about these scenarios are provided in Section 3 of the Supporting Information.

Regarding the CO_2_ from LEA combustion, CCS scenarios related to HDO and BD achieve the best performance in all indicators except for the biogeochemical nitrogen flow, with a PBF up to 4% lower than that of diesel in the equivalent GREEN_CCS_^M2040^ scenarios (i.e., ceteris paribus change). On the contrary, the absence of CCS or CCU after byproduct cogeneration can increase impacts on climate change up to 3.7 times compared to diesel (GRAY_NoCCU_^2020^ scenario). Moreover, in GRAY or YELLOW pathways disregarding the use of LEA, impacts on climate change can reach values 3.6 times higher than BAU, thus highlighting the importance of exploiting this byproduct through CCU or CCS strategies.

Utilizing the CO_2_ from cogeneration in the cultivation stage (CCU) results in higher CO_2_ emissions than storing that same CO_2_ in geological reservoirs (CCS), owing to the energy associated with CO_2_ recycling and the eventual release of the CO_2_ captured in the open ponds. The exception is the HTL-based scenario, which achieves lower CFP and PBF in CCU scenarios than that in CCS scenarios. This happens because the CO_2_ generated in the HTL process covers almost entirely the CO_2_ demand from the wood-microalgae mixture, which has a lower CO_2_ requirement compared to 100% microalgae-based biofuel. This, in turn, translates into less CO_2_ required from DAC or NGP to meet the total demand.

As shown in Figure 3, HTL scenarios achieve between 1 and 37% lower CFP than HDO scenarios when M2020 is considered. This is because the HTL process requires less energy and resources (i.e., due to the elimination of the drying and extraction process) than HDO, where no extraction stage is necessary. For HTL scenarios, we consider that the CO_2_ contained in the HTL residue would be released into the atmosphere, in a similar way as in the digestate slurry.^50^ Conversely, the CO_2_ in the gaseous phase can be used in both CCU and CCS processes. This CO_2_ flow from HTL represents 43% of the CO_2_ that can be recovered after LEA combustion. This lower CO_2_ availability makes the PBF of the HTL-GREEN_CCS_^M2040^ scenario 3% worse than that for the HDO-GREEN_CCS_^M2040^ one (4.86 vs 4.74, respectively).

Interestingly, the *ceteris paribus* change of the M2020 by a mix based on renewable sources (M2040) allows us to reduce the PBF of microalgae biofuels by up to 5% for HDO-GREEN_CCS_^M2040^. This represents a reduction in climate change impacts of 77% compared to the equivalent scenario based on M2020. Overall, changes in the electricity mix are most noticeable for HDO-GREEN_CCS_ scenarios than that for the other cases due to their higher demand for microalgae biomass that, in turn, leads to an increased electricity consumption during cultivation. For these scenarios, CO_2_ emissions and biomass use, associated with the 2020 electricity mix, result in higher impacts on climate change and nitrogen flows PBs. In the case of conventional biofuels, a change in the electricity mix would produce a very small improvement in the PBF: 0.1% for biodiesel and 1% for HDO. This happens because, unlike the microalgae-based fuels that strongly depend on the electricity matrix, most emissions from conventional biofuels occur during biomass cultivation, in processes related to land-use change, fertilizer use, agricultural materials, and transport.^11,51,52^ In addition, wind-powered scenarios are used here as a utopic scenario for the sake of comparison (see Section 3 in the Supporting Information). According to our results, the performance of the M2040 scenarios is very close to wind-based ones. As an example, the best scenario for biofuels using the M2040 mix (HDO-GREEN_CCS_^M2040^) shows a PBF only 0.6% higher than using wind.^53^

#### 3.1.3 Opportunities and Limitations of Biofuels from Microalgae

Microalgae-based fuels present an uneven performance, depending on the particular scenario assessed. In terms of climate change, the best-performing scenario corresponds to HDO-GREEN_CCS_^2040^, which only occupies 25% of the SOS, 77% less than the BAU scenario. In addition, this scenario does not transgress any of the other PB considered and shows the lowest impacts on the PBF, the CFP, and HH metrics.

Since road freight transport activities represent around 6% of the PBF of the whole economy, the value obtained for SOS of climate change by HDO-GREEN_CCS_^M2040^, which is four times lower than BAU, reflects a change in PBF of 4% (i.e., 4.94 for BAU vs 4.74 for HDO-GREEN_CCS_^M2040^).

At the other end of the spectrum, we find HDO-GRAY_NoCCU_^M2020^, which shows a transgression of the climate change PB 3.6 times larger than the BAU scenario (see Table S29 in the Supporting Information).

Despite the potential of certain biofuels to help the heavy-duty transport sector to operate within SOS, the increase of activities for microalgae biofuel production will lead to a rise in the use of resources that are currently used by other activities. For instance, satisfying the current energy demand of the heavy-duty transport sector using the lowest impact scenario (HDO-GREEN_CCS_^M2040^) would require approximately 18% of the world’s annual electricity consumption.^54,55^ This would require a CO_2_ removal capacity of 4.75Gt CO_2_, which is less than 1% of the global CO_2_ removal quota proposed for global warming mitigation.^56^ The situation is not better for conventional biofuels, considering that each tkm with HDO soybean requires 0.135 m^2^/yr: 29% of the world’s total cultivated area in 2019^57^ would be needed to cover the global demand for fuel for freight road activities.

### 3.2 Relative Contribution to Biosphere Integrity

**Figure 4:**
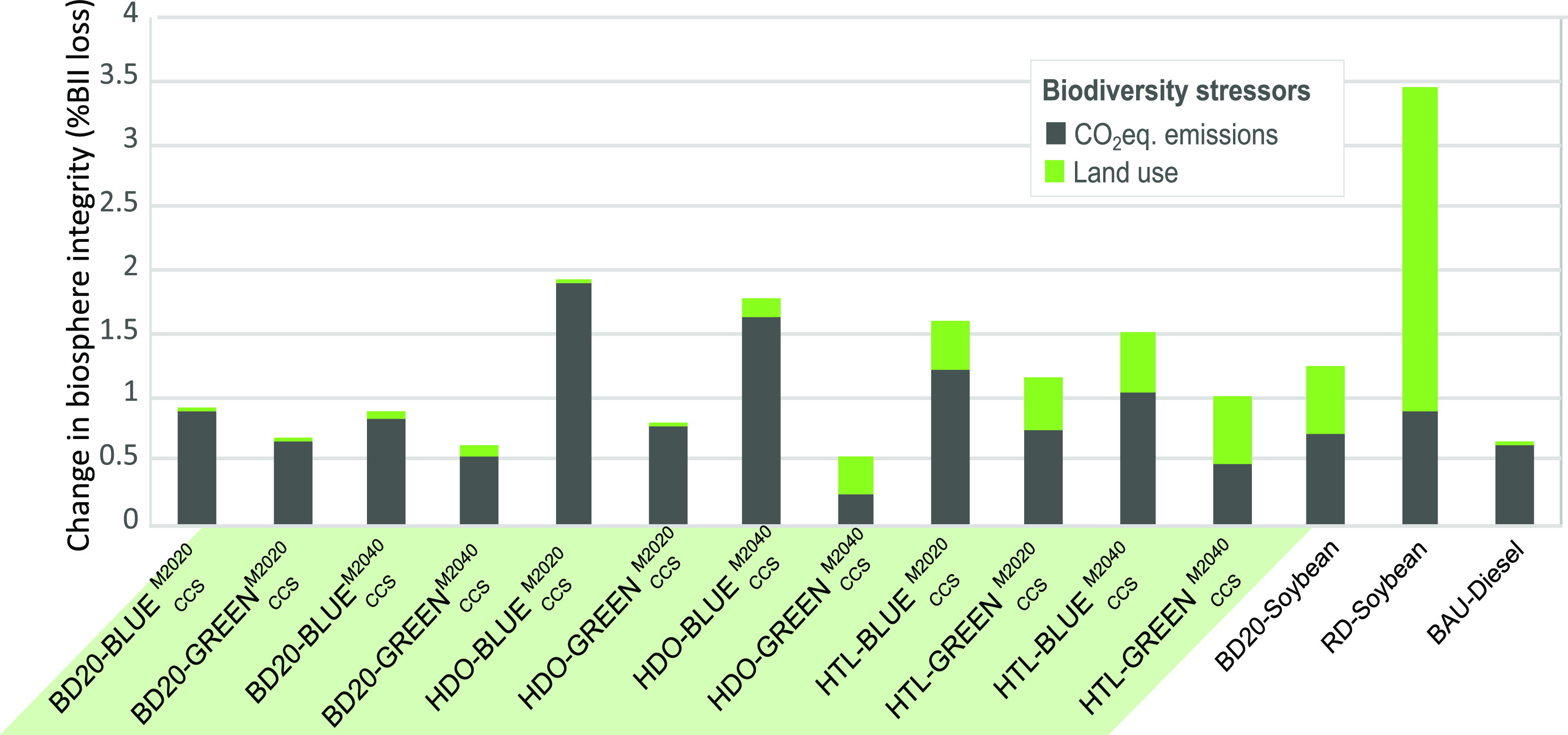
Impacts on change in biosphere integrity for different scenarios for the road freight activities, expressed as the mean species abundance loss caused by the two main stressors of biodiversity loss: LU (i.e., direct land use) and CO_2_ emissions.

One of the well-known drawbacks of conventional biofuels is the pressure exerted on land use, which, in turn, is a threat to biodiversity.^58^ Hence, while biofuels can reduce the CO_2_ emissions in transportation, which is one of the main stressors of biodiversity lost, this is typically counterbalanced by larger impacts in land use.^59^ On the other hand, biofuels from algae stand out for their high yield per ha that grants a low impact on land-system change (e.g., 50 t microalgae oil/ha yr vs 0.5 t soybean oil/ha yr).^11,60,61^ This translates into lower impacts on biosphere integrity compared with, e.g., soybean scenarios: up to 4.1 times lower considering the current electricity mix scenario (HDO-GREEN_CCS_^M2020^ vs HDO soybean) and 6.5 times lower considering the 2040 sustainable electricity mix scenario (HDO-GREEN_CCS_^M2040^), as shown in Figure 4. This difference becomes smaller for BD20, where benefits in reduced use of land (4.7 × 10^–5^ m^2^/yr tkm by diesel vs 9.6 × 10^–2^ m^2^/yr tkm by soybean HDO) are partially offset by the larger CO_2_ emissions from diesel, which constitutes 80% of the blend.

On the other hand, Figure 4 shows that the stressors of change in biosphere integrity for the case of microalgae-based biofuels come mainly from CO_2_ emissions, with very little contribution from land use. Unlike for diesel, these emissions are not due to the combustion process but mainly stem from the energy requirements of activities such as capturing the CO_2_ and recirculating water in open ponds. This is reflected by comparing equivalent scenarios with the *ceteris paribus* change of the electricity mix. As an example, the change in biosphere integrity attained by the HDO-GREEN_CCS_^M2040^ scenario is 0.5%, a 33% lower than when using the 2020 electricity mix (HDO-GREEN_CCS_^M2020^). From Figure 4, we also observe an increase in the land use stressor related to the increase in renewables for HDO-GREEN_CCS_^M2020^ scenario, contributing 59% to this impact. Renewable energies dominating the sustainable mix require a larger surface than non-renewables (up to 2.1·10^–3^ m^2^/W vs up to 5.1·10^–2^ m^2^/W).^62^

The only exception to this pattern is HTL, for which the land use stressor contributes between 34% and 55% of the total impact. This is due to the use of a 30% of wood pellets in the biomass mixture used with the microalgae. Although the contribution to this stressor could be reduced by modifying the share of wood in the mixture, this is the composition considered as technically and economically optimal in the literature due to the high HDO yields achieved and the current high costs of microalgae (around 590 USD/ton ash-free dry biomass).^63^

### 3.3 Relative Contribution to Human Health

**Figure 5:**
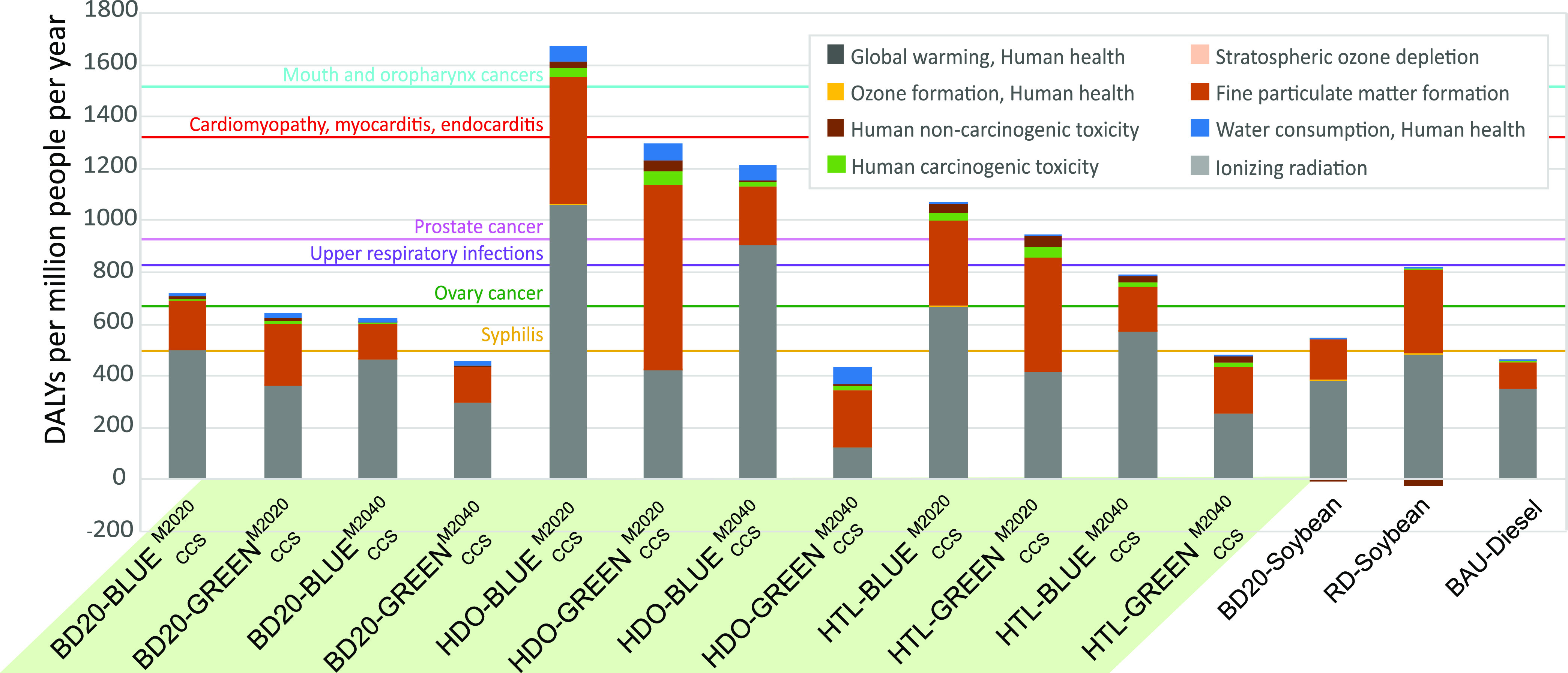
Impacts on human health for different scenarios for the road freight activities. Contribution of midpoints to the total impacts on human health, expressed in Disability-Adjusted Life Years (DALYs) per million people per year, based on global demand for road freight activities.

Even though the substitution of fossil fuels with biofuels aims mostly to mitigate climate change, both types of fuels still release harmful emissions such as particulate matter (PM), non-volatile organics (NVOC), or NO_*x*_^64,65^ during combustion. So far, we have discussed the implications of these emissions for the planet, but in this section, we will turn our attention to the consequences that direct (and indirect) emissions from the heavy-duty transport sector have on HH (Figure 5). Note that these results include the emissions generated during the combustion of the three types of fuels (HDO, BD20, and diesel) in the vehicle. The details for these emissions are shown in Tables S23–S25 in the Supporting Information.

For most scenarios, the main stressors for impacts on HH are global warming and PM, with marginal contributions from water consumption and carcinogens. Some microalgae-based scenarios, such as HDO-GREEN_CCS_^M2020^ have an impact on human health almost three times higher than diesel (Figure 5), despite achieving almost an equivalent PBF (Figure 3). This is because the emission of compounds such as PM has a different influence on some Earth’s climate system than that on human health, evidencing the need to carry out both analyses in parallel.

According to our study, and assuming the exclusive use of diesel (rightmost bar in Figure 5), we estimate global emissions from the heavy-duty transport sector to be approximately 3 Gt CO_2_ emissions/yr. The contribution of midpoints to the total impacts on human health from the diesel-based scenarios would cause 479 DALYs per million people per year. For the sake of comparison, it could be said that the health burden caused by the heavy-duty transport sector is similar in magnitude to the one caused by syphilis.

On the other hand, microalgae-based biofuels present very different impacts depending on the scenario, ranging from 454 to 2376 DALYs per million people per year. The largest impact corresponds to the HDO-GRAY_NoCCU_^M2020^ scenario (see Table S29). This scenario, whose performance on PBs is very similar to that of diesel, presents a three times higher impact on HH, achieving a similar magnitude to that of heart diseases such as cardiomyopathy.^44^ The reason behind such a large impact is the intensive use of energy from the current fossil-based mix, which generates emissions contributing to global warming and PM formation.

Interestingly, the *ceteris paribus* change of M2020 by the mix based on renewable (i.e., M2040) sources allows us to reduce the impact on HH to 454 DALYs for HDO-GREEN_CCS_^M2040^, i.e., 67% lower than HDO-GREEN_CCS_^M2020^ scenario and 5% lower than diesel. This places the HDO-GREEN_CCS_^M2040^ scenario as the one achieving the lowest impact on HH without increasing the impacts related to PBs.

## 4 Conclusions

This work compared 68 different biofuel production routes from microalgae for freight road transport under different scenarios considering a cradle-to-wheel perspective. We quantified the impact of these scenarios on seven Earth-system processes and on CFP and human health to assess the potential benefits of replacing conventional fossil fuels.

Given the current challenges facing electric vehicles, we believe that liquid fuels will continue to play a crucial role in the transportation sector, at least in the short to medium term, until challenges such as decarbonization of the electricity mix, battery energy densities, and refueling times can be effectively addressed.

We found that conventional fossil fuels for freight road transport (e.g., diesel) are unsustainable since they substantially transgress the climate change PB. In fact, the freight road transport sector alone already occupies 110% of the climate change PB, leaving no place for the remaining economic activities. At the same time, alternative routes based on microalgae could substantially improve the absolute environmental sustainability level of BAU, where six scenarios based on direct air capture, carbon capture and storage, and electricity mix projected for 2040 are particularly appealing and, in principle, could operate under PBs. In contrast, when the current 2020 electricity mix is considered, HTL scenarios perform better than HDO or BD20 alternatives due to the lower use of thermal energy achieved by avoiding the extraction stage.

Climate change boundaries show the largest share occupied by the transport sector due to their close link with fuel combustion and corresponding carbon emissions. Scenarios using fossil CO_2_ for microalgae growth and the M2020 electricity mix led to an increase in the PBF and HH impacts compared with diesel in all the scenarios. On the other hand, this study shows the importance of using carbon capture utilization and storage strategies after cogeneration with lipid-extracted algae to achieve scenarios that can operate within PBs. However, even in these cases, the use of a fossil-based electricity mix can increase HH impacts relative to the BAU scenario (e.g., 2.8 times in the case of HDO-GREEN_CCS_^M2020^).

Finally, although several scenarios of biofuel production from microalgae fail to operate within PBs, they still achieve six times lower change in biosphere integrity compared with first-generation biofuels, such as that based on soybean. This demonstrates not only the potential of microalgae to combat climate change but also highlights the opportunities for reducing impacts on the biosphere. The true potential of microalgae-based fuels to mitigate the impact on the Earth system processes depends on their geographic location, influenced by factors such as radiation and temperature, which affect microalgae growth and yield. In this contribution, we use harmonized data from 1400 microalgae production facilities scattered throughout the United States. This means that the potential of microalgae to reduce environmental impacts could increase or decreased in other specific locations.

In any case, fuel production alternatives and their effects should be considered under a holistic approach to analyze the broad range of potential implications. In our study, we found that it is possible for the heavy-duty transport sector to operate within PBs while keeping impacts on HH lower than that in the BAU scenario. However, algae-based fuels would require approximately 18% of the current global electricity consumption.

Promoting microalgae biofuels today as an interim solution for the transport sector could be better than using traditional biofuels as the former can outperform the latter in terms of CO_2_ emissions and changes in biosphere integrity. Combined with carbon capture and storage, these plants could remove CO_2_ from the atmosphere, which is essential to achieving net-zero targets. In this context, PBs metrics, such as the one presented in this contribution, provide a robust framework for holistic assessments with the ability to minimize burden-shifting and help policymakers develop better-informed policies.

## Supporting Information Available

Modeling details for the pathways modeled, life cycle inventories, additional details on the PB methodology, and environmental and assessment of all scenarios (PDF)

## Abbreviations

AESA: absolute environmental sustainability assessment
BAU: business-as-usual
BD: biodiesel
BD20: biodiesel blending with diesel in a 20% v/v
BG: CO_2_ flow as emissions from background activities
CBF: CO_2_ flow as emissions from biofuel combustion
CCS: carbon capture and storage in geological reservoirs
CCU: carbon capture and utilization
CFEED: CO_2_ flows for microalgae growth
CFP: carbon footprint
CLEA: CO_2_ flow emissions from carbon embedded on lipid-extracted algae
DAC: direct air capture
DALYs: disability-adjusted life years
GHG: greenhouse gas emissions
HDO: hydrodeoxygenation
HH: human health
HLS: CO_2_ losses from algae cultivation
HTL: hydrothermal liquefaction
LCA: life cycle assessment
LCIs: life cycle inventories
LEA: lipid-extracted algae
M2020: 2020 global electricity mix
M2040: sustainable global mix for 2040
NGP: natural gas power plant
PBs: planetary boundaries
PBF: planetary boundary footprint
SOS: safe operating space for humanity
TRL: technology readiness levels
