# Supplementary material for: Microalgae Biofuel for a Heavy-Duty Transport Sector within Planetary Boundaries
Source: ACS Sustain Chem Eng. 2023 Jun 13;11(25):9359–71. doi: 10.1021/acssuschemeng.3c00750 (PMC10302904; doi:10.1021/acssuschemeng.3c00750)
Supplement: Supplementary file 1 — sc3c00750_si_001.pdf [file sc3c00750_si_001.pdf]

Supporting Information

The implications of microalgae biofuel production for the heavy-duty transport sector under planetary boundary perspective

Richard Cabrera-Jiménez†, Victor Tulus‡, Jordi Gavaldà†, Laureano Jiménez†, Gonzalo Guillén-Gosálbez*‡, Carlos Pozo*†

† Departament d'Enginyeria Quimica, Universitat Rovira i Virgili, Av. Països Catalans 26, 43007 Tarragona, Spain.

‡ Institute for Chemical and Bioengineering, Department of Chemistry and Applied Biosciences, ETH Zürich, Vladimir-Prelog-Weg 1, 8093 Zürich, Switzerland.

Number of pages: 28

Number of figures: 8

Number of tables: 32

This document is structured in 3 parts, section 1 provides details about the scenarios, and planetary boundaries (PB) addressed in this paper, section 2 describes the data sources and the LCI developed for the analysis, and section 3 presents additional results not shown in the manuscript for the sake of shortness.

1. Definition of scenarios and planetary boundaries

The scenarios considered in the analysis and the corresponding labels are provided in **Table S1**. In **Table S2,** the abbreviations adopted for the PBs are described, and Table S3 provides the control variables values, along with the proposed boundaries.

Table S1. Correspondence between labels and scenarios reported in this study.

Label	Scenario
BAU	Business-as-usual (global demand for road freight covert by diesel)
M2020	Current global electricity grid mix.[1]
M2040	2040 sustainable development (SD) grid mix. [2]
NGP	Carbon dioxide provided to algae harvesting is captured from the natural gas power plant.[3]
DAC	Carbon dioxide provided to algae harvesting is captured from direct air capture plant.[4]
CCS	Carbon dioxide after cogeneration is captured and stored in a geological reservoir.[5,6]
CCU	Carbon dioxide after cogeneration is captured and send to algae harvesting. [7]
NoCCU	Remaining Lipid Extracted Algae (LEA) is not used as a source of energy, therefore the use of CO_2 is not considered and this is considered strictly as an emission
C	Cogeneration of energy occurs through the direct combustion of LEA to supply heat via a steam boiler. Considering an electric efficiency of 21.7% and a heat recovery efficiency of 65% and a biomass moisture of 20%[7]
B	Cogeneration of energy is carried out through anaerobic digestion to produce biogas and its subsequent use in a gas turbine. Considering an electric efficiency of 33% and a heat recovery efficiency of 64%. [7]
ACR	Combustion gases from the cogeneration process are released into the

	atmosphere without carbon dioxide capture
HDO	Biofuel from hydrodeoxygenation
BD20	Biofuel from transesterification in a blend with fossil fuels 20% vol
HTL	Biofuel from hydrothermal liquefaction

†HTL-Soybean is not considered because for biofuel production by HTL a moisture content of 20% is considered. [8]. Soybean oil is considered a feedstock and currently the soybean residue has an important economic value (i.e., 56% allocation). Therefore, the use of whole grains as feedstock is not considered. [9]

Figure S1 shows in a more detailed representation than Figure 3 of the manuscript, showing the interactions between the different processes in the scenarios under analysis.

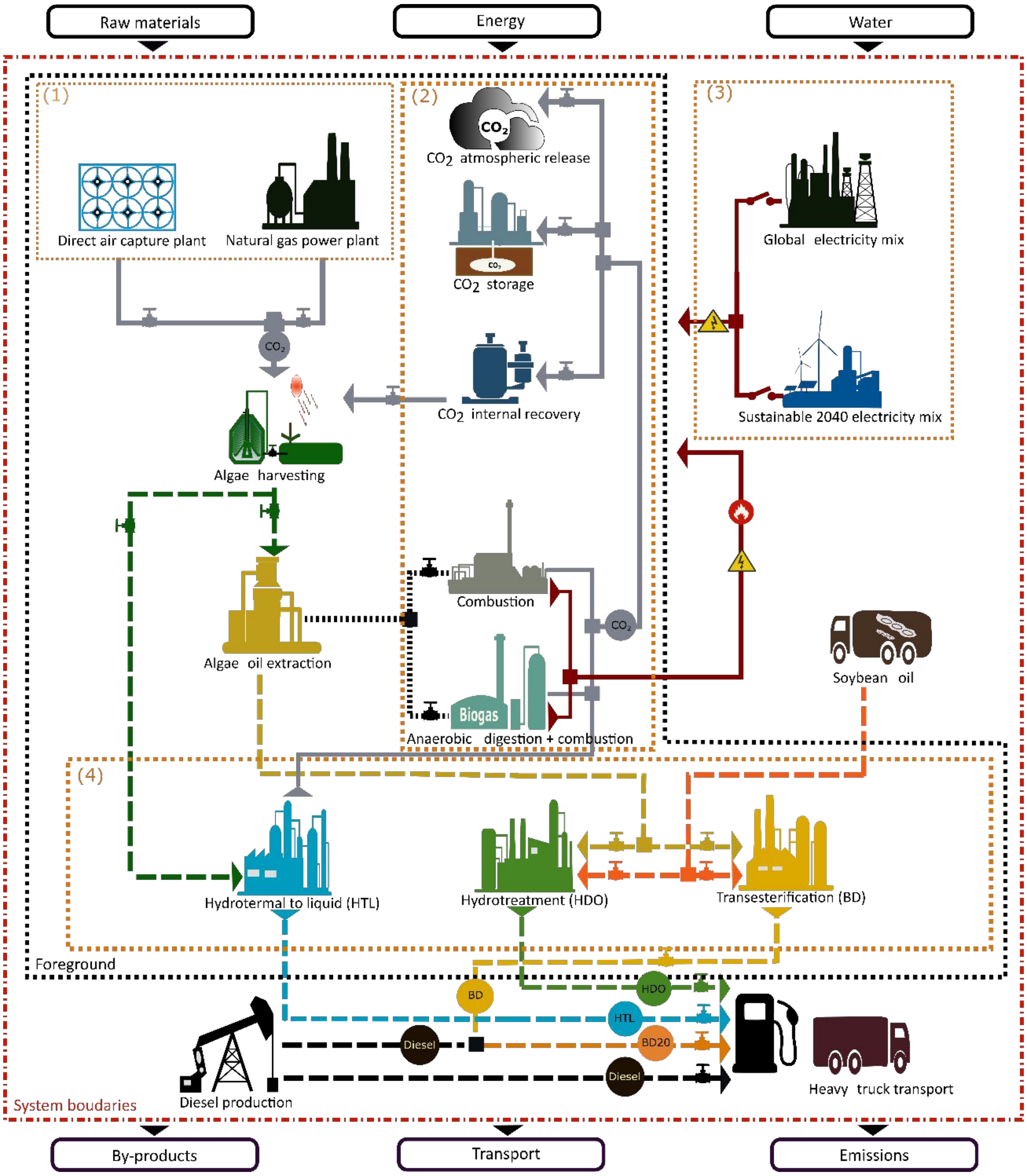

Figure S1. Detailed conceptual framework for biofuels-from microalgae considering 71 scenarios: (1) Carbon feedstock from carbon captured from DAC and power plant; (2) Carbon storage, carbon utilization, and carbon

emissions as end life of CO_2 from lipid extracted algae (LEA); (3) Electricity from wind, current global electricity mix, and sustainable mix; (4) Transesterification, hydrotreatment and hydrothermal to liquid as the biofuel conversion process.

Table S2. Abbreviations adopted to denote the control variables associated with the investigated Planetary Boundaries.

Label	Planetary boundary or earth system process	Control variable
aCO2	Climate change	CO_2 atmospheric concentration [ppm]
EI	Climate change	Energy imbalance [W m^{-2}]
SOD	Stratospheric ozone depletion	Stratospheric ozone concentration [Dobson units, DU]
OA	Ocean acidification	Carbonate ion concentration [Ω_{arag}]
P	Biogeochemical flow	Phosphorus flow from freshwater systems into the ocean [Tg P a^{-1}]
N	Biogeochemical flow	Industrial and intentional biological fixation of N [Tg N a^{-1}]
LSC	Land system change	Area of forested land [% of original forest cover]
FWU	Freshwater use	Maximum amount of consumptive blue water use [km^3 a^{-1}]
CBI	Terrestrial biosphere integrity	Functional diversity [Biodiversity Intactness Index, BII]

Table S3 Control variables values, along with the proposed boundaries considering the whole economy.

**Label**	**Unit**	**Planetary boundary**	**Zone of uncertainty**	**Natural background level**	**Safe operating space**	**Current anthropogenic status**
aCO2	ppm CO_2	350	350 - 450	278	72	398.5
EI	W m^{-2}	1	1-1.5	0	1	2.3
SOD	DU	275	275 -261	290	15	200
OA	Ω_{arag}	2.75	2.75-2.4	3.44	0.69	2.89
P	Tg P $year^{-1}$	11	11-100	1.1	9.9	22
N	Tg N $year^{-1}$	62	62-82	0	62	150
LSC	%	75	75-54	100	25	62
FWU	km^3 $year^{-1}$	4000	4000-6000	0	4000	2600
CBI	BII loss	10	10-70	0	10	26.8

2. Data sources and the LCI developed

2.1. Data sources

The required data used in the LCA calculations is provided in this section. Most values were taken from the GREET database, [7] which contains harmonized data from 1400 facilities with different annual capacities, as shown in Table S4.

Table S4. Annual capacity of plants considered for the LCI data gathered.

Facility	Annual capacity	Reference
Microalgae cultivation pond	350 ton dry algae	[10]
Microalgae cultivation facility	1750 ton dry algae	[10]
Biofuel plant	42500 ton diesel	[11]
CO_2 for direct air capture	1.08 ton CO_2	[4]
CO_2 capture and storage in a geological reservoir	8.7 ton CO_2	[6]

Table S5 to Table S21 provide the different life cycle inventories considered for microalgae biofuel production, along with all the dependent sub-processes that were adapted from the original references. The final LCIs were obtained by combining data from the process model in GREET with information from the literature and Ecoinvent v3.7. [1] The latter activities, missing in Ecoinvent and requiring a tailored intermediate inventory analysis based on the literature, are labeled with a "*" detailed in LCI Tables. Furthermore, as seen in tables S5 and S21, several of the inputs to the foreground system were modeled utilizing additional information from the literature and Ecoinvent.

The "Electricity" activity differs depending on the scenario. Table S22 and Table S23 provide a thorough breakdown of the various electricity scenario options. We assume that the electricity consumed in the background system is modeled with the default activities in Ecoinvent v3.7. Hence, we only change the electricity of the foreground system while keeping the energy generation activities in the background system unaltered.

Table S5-S7 provide information about microalgae production with a moisture of 20%. The process considers the use of open pools, where water and fertilizers are recirculated after the filtration and drying process so that the replacement water introduced corresponds to the water lost by evaporation and blowdown to avoid excess mineral and salt build-up, and to regulate the pH of the culture medium. [12] The harvesting and drying process is carried out in two stages using flocculation followed by mechanical filtration using centrifuges [7].

The yield of microalgae is influenced by their geographical location and, as a result, the potential of microalgae to reduce their impacts on seven Earth-system processes can vary across different scenarios. The yield of microalgae varies widely across the world, ranging from 4.43 $g/m^2/day$ (1.13 m^3 oil/ha/yr) to 38.80 $g/m^2/day$ (27 $m^3/ha/yr$), with the geographical location and season affecting growth due to variations in radiation and temperature. [13] However, for the purposes of this study, harmonized values corresponding to 26.37 $g/m^2/day$ (3.6 $m^3/ha/yr$) were used, based on studies conducted in the United States region. [10]

The process of CO_2 capture from direct air capture (Table S15) or natural gas power plant (Table S15) provides concentrations higher than 97%. This concentration is adopted since GREET describes a pure CO_2 flow for microalgae growing. Furthermore, in the case of CO_2 from the natural gas power plant to remove compounds such as nitrogen oxides, sulfur oxides and heavy metals that can adversely affect the growth of microalgae.

Table S5. LCI of the foreground system microalgae production based on open ponds excluding the recovery of CO_2 after energy cogeneration based on lipid extracted algae (LEA).

**Process**	Microalgae production w/o CO_2 from downstream activities		
	**Ecoinvent entry**	**Description**	**Amount**
Inputs:			
	Market for electricity medium voltage	Electricity cultivation and drying [kWh]	0.614
	Nutrient supply from urea, ROW	Nitrogen fertilizer [kg]	0.025
	Market for sodium phosphate glo	Phosporus fertilizer [kg]	0.010
	Aluminium sulfate, powder//[ROW] market for aluminium sulfate, powder	Flocculant [kg]	0.004
	Concrete block//[RoW] market for concrete block	Concrete [kg]	0.023
	Market for reinforcing steel	Steel [kg]	0.001
	Extrusion, plastic film//[GLO] market for extrusion, plastic film	Plastic [kg]	0.005
	Cast iron//[GLO] market for cast iron	Cast iron [kg]	0.003
	Water, unspecified natural origin, RoW	Water lost to blowdown and evaporation [kg]	57.3
	Occupation, unspecified, in ground	Land occupation [m^2a]	0.00013
	*CO_2 from NG or DAC	CO_2 input [kg]	2.67
Outputs:			
		Microalgae biomass 20% moisture[kg]	1
		CO_2 emmisions [kg]	0.459

*CO_2 from NG and DAC are the possible activities that supply CO_2 to the process and are detailed on Table S15 and Table S16, respectively. The LCI data for microalgae biomass production are retrieved from the GREET2022 datasheet in section 1.3 of the "Algae" category. [14] More information and details on cultivation can also be found in the document "2017 Algae Harmonization Study". [10]

Table S6. LCI of the foreground system microalgae production based on open ponds with CO_2 recovery after LEA combustion for energy cogeneration.

Process:	Microalgae production w CO_2 from LEA combustion		
	**Ecoinvent entry**	**Description**	**Amount**
Inputs:			
	Market for electricity medium voltage	Electricity Cultivation and drying [kWh]	0.293
	Nutrient supply from urea, ROW	Nitrogen fertilizer [kg]	0.025
	Market for sodium phosphate [GLO]	Phosporus fertilizer [kg]	0.010
	Aluminium sulfate, powder//[ROW] market for aluminium sulfate, powder	Flocculant [kg]	0.004
	Concrete block//[RoW] market for concrete block	Concrete [kg]	0.023
	Market for reinforcing steel	Steel [kg]	0.001
	Extrusion, plastic film//[GLO] market for extrusion, plastic film	Plastic [kg]	0.005
	Cast iron//[GLO] market for cast iron	Cast iron [kg]	0.003
	Water, unspecified natural origin, RoW	Water lost to blowdown and evaporation [kg]	57.3
	Occupation, unspecified, in ground	Land occupation [m^2a]	0.00013
	*CO_2 from NG or DAC	CO_2 input [kg]	1.229
Outputs:			
		Microalgae biomass 20% moisture [kg]	1
		CO_2 emissions [kg]	0.459

†For CO_2 reuse from LEA combustion, 1.23 kg CO_2/kg algae biomass is supplied, 0.336 kWh of electricity is delivered from the cogeneration plant, and 0.015kWh are required for capture and transport of CO_2 from LEA combustion with a moisture of 20%. The LCI data for microalgae biomass production are retrieved from the GREET2022 datasheet in section 1.3 of the "Algae" category.[14] More information and details on cultivation can also be found in the document "2017 Algae Harmonization Study".[10] The electric and heat recovery efficiencies are 21.7% and 65%, respectively.[7,15]

*CO_2 from NG and DAC are the possible activities that supply CO2 to the process and are detailed in Table S14 and Table S15, respectively.

Table S7. LCI of the foreground system microalgae production based on open ponds with CO_2 recovery after LEA combustion for energy cogeneration.

Process:	Microalgae production w CO_2 from biogas combustion		
	**Ecoinvent entry**	**Description**	**Amount**

Inputs:			
	Market for electricity medium voltage	Electricity cultivation and drying [kWh]	0.0368
	Nutrient supply from urea, ROW	Nitrogen fertilizer [kg]	0.025
	Market for sodium phosphate glo	Phosporus fertilizer [kg]	0.010
	Aluminium sulfate, powder//[ROW] market for aluminium sulfate, powder	Flocculant [kg]	0.004
	Concrete block//[RoW] market for concrete block	Concrete [kg]	0.023
	Market for reinforcing steel	Steel [kg]	0.001
	Extrusion, plastic film//[GLO] market for extrusion, plastic film	Plastic [kg]	0.005
	Cast iron//[GLO] market for cast iron	Cast iron [kg]	0.003
	Water, unspecified natural origin, RoW	Water lost to blowdown and evaporation [kg]	57.3
	Occupation, unspecified, in ground	Land occupation [m^2a]	0.00013
	*CO_2 from NG or DAC	CO_2 input [kg]	1.77
Outputs:			
		Microalgae biomass 20% moisture [kg]	1
		CO_2 emmisions [kg]	0.459

For CO_2 reuse from biogas combustion, 0.69 kg of CO_2 are supplied for each kg of algae introduced to the microalgae production process, 0.592 kWh of electricity are delivered from the cogeneration plant, and 0.008 kWh are required for capture and transport of CO_2 from biogas combustion. The electric and heat recovery efficiencies are 33.1% and 64%, respectively for combined heat and power through a gas turbine. [7,15,16].

*CO_2 from NG and DAC are the possible activities that supply CO_2 to the process and are detailed on table Table S15 and Table S16, respectively.

Table S8. LCI of the microalgae oil production based on wet extraction without cogeneration.

**Process:**	Microalgae oil extraction		
	**Ecoinvent entry**	**Description**	**Amount**
Inputs:			
	*Algae biomass 20% moisture	Algae biomass 20% moisture [kg]	4.678

	Heat, central or small-scale, other than natural gas {GLO}| market group for | APOS, U	Thermal energy for extraction [MJ]	11.124
	Electricity, medium voltage {GLO}| market group for | APOS, U	Electricity for extraction [kWh]	0.927
	Hexane {GLO}| market for | APOS, U	Hexane [kg]	5.50E-05
Outputs:			
		Lipid-extracted biomass to recovery step [kg]	3.98
		Hexane emissions [kg]	5.50E-05
		Microalgae oil [kg]	1

The data for microalgae extraction are retrieved from the GREET2022 datasheet in section 2.3 from oil extraction column, of the "Algae" category. [14]

Table S9. LCI of the microalgae oil production based on wet extraction.

**Process:**	Microalgae oil extraction		
	**Ecoinvent entry**	**Description**	**Amount**
Inputs:			
	*Algae biomass 20% moisture	Algae biomass 20% moisture [kg]	4.678
	Heat, central or small-scale, other than natural gas {GLO}| market group for | APOS, U	Thermal energy for extraction [MJ]	0
	Electricity, medium voltage {GLO}| market group for | APOS, U	Electricity for extraction [kWh]	0.927
	Hexane {GLO}| market for | APOS, U	Hexane [kg]	5.50E-05
Outputs:			
		Lipid-extracted biomass to recovery step [kg]	3.98
		Hexane emissions [kg]	5.50E-05
		Microalgae oil [kg]	1

†The heat demand is considered 0 for the scenarios with cogeneration of energy by direct combustion of LEA or cogeneration from biogas. In the first case, 23.12 MW/kg oil, and in the second case, 17.75 MW/ kg oil of thermal energy are generated [7].

Table S10. LCI of the energy cogeneration from lipid extracted algae combustion.

**Process:**	Energy cogeneration from LEA combustion		
	**Ecoinvent entry**	**Description**	**Amount**
Inputs:			
	Lipid-extracted biomass to recovery step	Lipid-extracted biomass [kg]	3.98

Outputs:			
		Thermal energy recovery [MJ]	23.12
		Electricity recovery [kWh]	2.73
		CO_2 recovered [kg]	5.76
		CO_2 emissions [kg]	1.265

LCI data for LEA combustion are retrieved from the GREET2022 datasheet in section 2.1 of the "Algae" category where 100% LEA to combustion is selected. [14]

Table S11. LCI of the energy cogeneration from biogas combustion.

**Process:**	Energy cogeneration from biogas		
	**Ecoinvent entry**	**Description**	**Amount**
Inputs:			
	*Lipid-extracted biomass	Lipid-extracted biomass [kg]	3.98
Outputs:			
		Thermal energy recovery [MJ]	17.75
		Electricity recovery [kWh]	3.81
		CO_2 recovered [kg]	2.33
		Anaerobic digestate residue [kg]	1.99
		Methane emissions [g]	16.96

LCI data for LEA combustion are retrieved from the GREET2022 datasheet in section 2.1 of the "Algae" category where 100% LEA to biogas combustion is selected. [14]

Table S12. LCI of the HDO100 from microalgae production.

**Process:**	HDO100 production from microalgae		
	**Ecoinvent entry**	**Description**	**Amount**
Inputs:			
	Electricity, medium voltage {GLO}| market group for | APOS, U	Electricity [kWh]	0.086
	*Microalgae oil	Microalgae oil [kg]	0.962
	Tap water {Europe without Switzerland}| tap water production, conventional treatment | APOS, U	Water [kg]	0.0077
	Hydrogen, liquid {RER}| market for | APOS, U	Hydrogen [kg]	0.031
Outputs:			
		HDO [kg]	1
		Fuel gas [kg]	0.253
		Heavy oil [kg]	0.175

LCI data for HDO production are retrieved from the GREET2022 datasheet in section 2.2 of the "Algae" category where “Renewable Diesel I” column is selected. [14]

†The economic allocation considered was 89% for HDO, 6% for fuel gas, and 5% for heavy oil.

Table S13. LCI of the BD100 from microalgae production.

**Process:**	BD100 production from microalgae		
	**Ecoinvent entry**	**Description**	**Amount**
Inputs:			
	Heat, central or small-scale, other than natural gas {GLO}| market group for | APOS, U	Thermal energy [MJ]	1.07
	Electricity, medium voltage {GLO}| market group for | APOS, U	Electricity [kWh]	0.0335
	Tap water {Europe without Switzerland}| tap water production, conventional treatment | APOS, U	Water [kg]	0.0076
	Methanol {GLO}| market for | APOS, U	Methanol [kg]	0.109
	Sodium methoxide {GLO}| market for | APOS, U	Sodium methoxide [kg]	0.0040
	Neutralising agent, sodium hydroxide-equivalent {GLO}| market for | APOS, U	Sodium hydroxide [kg]	0.0011
	Phosphoric acid, industrial grade, without water, in 85% solution state {GLO}| market for | APOS, U	Phosphoric acid [kg]	0.0004
	Hydrochloric acid, without water, in 30% solution state {RoW}| market for | APOS, U	Hydrochloric acid [kg]	0.0017
	Sulfuric acid {RoW}| market for sulfuric acid | APOS, U	Sulfuric acid [kg]	0.0011
	*Microalgae oil	Microalgae oil [kg]	0.958
Outputs:			
		Biofuel [kg]	1
		Glycerin [kg]	0.097

LCI data for biodiesel production are retrieved from the GREET2022 datasheet in section 2.2 of the "Algae" category where “Biodiesel” column is selected. [14]

†Economic allocation of 95.7% for biofuel and 4.3% for glycerin were considered.

Table S14. LCI of the HTL from microalgae production.

**Process:**	HTL100 production from microalgae		
	**Ecoinvent entry**	**Description**	**Amount**

Inputs:			
	Heat, district or industrial, natural gas {GLO}| market group for | APOS, U	Heat [MJ]	8.516
	Electricity, medium voltage {GLO}| market group for | APOS, U	Electricity [kWh]	0.190
	Tap water {Europe without Switzerland}| tap water production, conventional treatment | APOS, U	Water [kg]	0.647
	Wood chips, dry, measured as dry mass {RoW}| market for | APOS, U	Wood [kg]	0.642
	*Microalgae biomass	Microalgae biomass [kg]	1.613
	Sulfuric acid {RER}| market for sulfuric acid | APOS, U	Sulfuric acid [kg]	0.076
	*HTL catalyst	Catalyst [kg]	0.000122
Outputs:			
		HTL [kg]	0.673
		Gasoline [kg]	0.327
		CO_2 emissions [kg]	2.495

LCI data for HTL production are retrieved from the GREET2022 datasheet in section 2.2 of the "Algae" category where “Hydrothermal liquefaction” column is selected. [14] More information and details on cultivation can also be found in the document "2017 Algae Harmonization Study".[10]

*LCI of catalyst are detailed in Table S21.

†Economic allocation of 67.3% for HTL and 32.7% for gasoline were considered.

Table S15. LCI of the CO_2 from direct air capture plant. [4]

**Process:**	CO_2 from direct air capture plant		
	**Ecoinvent entry**	**Description**	**Amount**
Inputs:			
	Heat, district or industrial, natural gas {GLO} | market group for	Natural gas [MJ]	5.25
	Electricity, high voltage {GLO} | market group for	Electricity [kWh]	0.234
	Tap water {GLO} | market group for	Water [kg]	3.11
	Calcium carbonate, precipitated {RoW} | market for calcium carbonate, precipitated	Calcium carbonate [kg]	0.0199
Outputs:			
		CO_2 captured [kg]	1

LCI data for CO_2 capture from direct air capture plant was modeled in Ecoinvent based on the data provided by Keith et al.[4]

Table S16. LCI of the CO_2 capture from natural gas power plant.

Process:	CO_2 capture from natural gas power plant		
	**Ecoinvent entry**	**Description**	**Amount**
Inputs:			
	Natural gas, from high pressure network (1- 5 bar), at service station {GLO} | market for	Natural gas [kg]	4.78E-01
	Water, decarbonized, at user {RER} | water production and supply, decarbonized	Water [kg]	3.30E-01
	Water, unspecified natural origin {GLO}	Water [kg]	1.45E-03
	*Catalyst	Catalyst [kg]	2.97E-06
	Monoethanolamine {GLO} | market for	Solvent MEA [kg]	8.77E-03
Outputs:			
		Carbon dioxide [kg]	1
		Electricity [kWh]	3.28
		CO_2 emissions [kg]	3.29E-01
		Monoethanolamine emissions [kg]	3.52E-03
		Nitrogen oxides [kg]	1.58E-03

†LCI of catalyst are detailed in Table S20. LCI data for CO_2 captured form a natural gas power plant was modeled in Ecoinvent based on the data provided by Ioannou et al. [17] The electricity shown is equal to the required demand minus the electricity for compression because compression at 30 bar is not required. [17,18] An economical allocation factor of 81% for electricity and 19% for CO_2 was considered. [3,19]

Table S17. LCI of a pipeline for supercritical CO_2 transport. [6]

Process:	Pipeline supercritical CO_2		
	**Ecoinvent entry**	**Description**	**Amount**
Inputs:			
	Occupation, construction site	Land occupation [m^2a]	3330
	Transformation from forest	Land trasnformation [m^2]	2000
	Transfformation, to heterogeneous, agricultural	Land transformation [m^2]	2000
	Water, unspecified natural origin/m3	Water [m^3]	187
	Sand {GLO}| market for	Sand [kg]	4.40E+06
	Diesel, burned in building machine {GLO}	Diesel [MJ]	3.31E+06
	Steel, low-alloyed {GLO}| market for	Steel [kg]	2.70E+05
	Drawing of pipe, steel {RER}| processing	Drawing of pipelines [kg]	2.70E+05
	Stone wool, packed {CH}| stone wool production, packed	Rock wool [kg]	5119
	Transport, helicopter {GLO}| market for | APOS, U	Transport, helicopter [hr]	26
	Transport, helicopter, LTO cycle {GLO}| market for | APOS, U	Transport helicopter [p]	10.4
	Transport, freight, lorry 16-32 metric ton, euro6 {RER}| market for transport, freight, lorry 16-32 metric ton, EURO6	Transport lorry [tkm]	3.15E+05

	Transport, freight train {RER}| market group for transport, freight train | APOS, U	Transport rail [tkm]	5.51E+04
	Disposal, inert waste 5% water to inert material landfill	Disposal of inert waste [kg]	4.40E+06
	Inert waste, for final disposal {CH}| treatment of inert waste, inert material landfill | APOS, U	Disposal, steel [kg]	1.35E+05
	Waste mineral wool, for final disposal {CH}| treatment of waste mineral wool, inert material landfill | APOS, U	Disposal, mineral wool [kg]	5.12E+03
Outputs:			
		Pipeline supercritical [km]	1

LCI data for the pipeline construction to transport CO_2 for geological storage was modeled in Ecoinvent based on the data provided by Wildbolz et al. [6].

Table S18. LCI for CO_2 transport through pipeline [6].

**Process:**	Storage CO2 aquifer 200 km pipeline		
	**Ecoinvent entry**	**Description**	**Amount**
Inputs:			
	*Well double aquifer	well double aquifer [p]	2.54E-11
	*CO_2 transport	CO_2 transport [km]	6.34E-9
Outputs:			
		CO_2 stored [kg]	1

LCI data for the storage of CO_2 transported in a well double aquifer was modeled in Ecoinvent based on the data provided by Wildbolz et al. [6]

Table S19. LCI for CO_2 capture in a geological reservoir [6]

**Process:**	CO_2 capture in a well double aquifer		
	**Ecoinvent entry**	**Description**	**Amount**
Inputs:			
	Occupation, industrial area	Industrial area [m^2a]	900
	Occupation, industrial area, vegetation	Vegetation occupation [m^2a]	8100
	Transformation, from pasture and meadow	Land transformation [m^2]	600
	Transformation, to industrial area	Land transformation [m^2]	60
	Transformation, to industrial area, vegetation	Land transformation [m^2]	540
	Deep well, drilled, for geothermal power {GLO}| market for | APOS, U	Drilling [m]	3.60E+03
	Cement, unspecified {CH}| market for cement, unspecified | APOS, U	Cement [kg]	1.26E+05
	Gravel, crushed {CH}| production | APOS, U	Gravel [kg]	1.32E+06
	Transport, freight, lorry 16-32 metric ton, euro6 {RoW}| market for transport, freight, lorry 16-32 metric ton, EURO6 | APOS, U	Transport lorry [tkm]	2.89E+04
	Transport, freight train {GLO}| market group for | APOS, U	Transport train [tkm]	1.26E+04
Outputs:			
		Well double aquifer [p]	1

LCI data for the use of a geological reservoir as CO_2 storage site was modeled in Ecoinvent based on the data provided by Wildbolz et al. [6].

Table S20. LCI of CO_2 capture and compression plant.

Process:	CO_2 capture and compression for storage		
	**Ecoinvent entry**	**Description**	**Amount**
Inputs:			
	Monoethanolamine {GLO}| market for | APOS, U	MEA [kg]	3.30E-04
	Water, decarbonised, at user {GLO}| market for | APOS, U	Water [kg]	6.26E-01
	Electricity, medium voltage {GLO}| market group for | APOS, U	Pumping [kWh]	3.46E-02
	Electricity, medium voltage {GLO}| market group for | APOS, U	Compression [kg]	9.24E-02
	Heat, district or industrial, natural gas {GLO}| market group for | APOS, U	Heating [MJ]	7.52E+00
	Storage CO2 aquifer 200 km pipeline	Storage [kg]	1
Outputs:			
		CO_2 capture [kg]	1.00E+00
		CO_2 emissions [kg]	3.23E-03
		H_2O emissions [kg]	1.21E+00
		O_2 emissions [kg]	8.27E-01
		N_2 emissions [kg]	4.44E+00
		MEA emissions [kg]	1.17E-04

LCI data for CO_2 capture and compression plant was modeled in Ecoinvent based on the data provided by Bello et al. [5]

†For electricity inputs GLO electricity is going to be changed to electricity M2040.

Table S21. LCI of catalyst

Process:	Catalyst		
	**Ecoinvent entry**	**Description**	**Amount**
Inputs:			
	Cobalt {GLO}| market for | APOS, U	Cobalt oxide [kg]	0.045
	Molybdenum trioxide {GLO}| market for | APOS, U	Molydnenum trioxide [kg]	0.145
	Aluminium oxide {GLO}| market for | APOS, U	Alumina [kg]	0.81
Outputs:			
		Catalyst [kg]	1

LCI of catalyst was modeled in Ecoinvent based on the data provided by Albrecht et. Al, [20] which studies the catalytic hydrotreating and hydrothermal liquefaction of microalgae.

Table S22. LCI of sustainable 2040 electricity mix. [2]

Process:	M2040 electricity mix		
	**Ecoinvent entry**	**Description**	**Amount**
Inputs:			
	Electricity, high voltage {RoW}| electricity production, hard coal | APOS, U	Energy from coal [kWh]	0.0092
	Electricity, high voltage {RoW}| electricity production, oil | APOS, U	Energy from oil [kWh]	0.0018
	Electricity, high voltage {RoW}| electricity production, natural gas, conventional power plant | APOS, U	Energy from natural gas [kWh]	0.103
	Electricity, high voltage {RoW}| electricity production, nuclear, pressure water reactor | APOS, U	Energy from nuclear source [kWh]	0.1598
	Electricity, high voltage {RoW}| electricity production, hydro, run-of-river | APOS, U	Energy from hydropower [kWh]	0.1583
	Electricity, high voltage {RoW}| heat	Energy from biomass	0.0885

	and power co-generation, wood chips, 6667 kW, state-of-the-art 2014 | APOS, U	[kWh]	
	Electricity, high voltage {RoW}| electricity production, wind, 1-3MW turbine, onshore | APOS, U	Energy from wind [kWh]	0.3572
	Electricity, high voltage {RoW}| electricity production, deep geothermal | APOS, U	Energy from geothermal [kWh]	0.0099
	Electricity, low voltage {RoW}| electricity production, photovoltaic, 570kWp open ground installation, multi-Si | APOS, U	Energy from photovoltaic [kWh]	0.1038
	Electricity, high voltage {RoW}| electricity production, solar thermal parabolic trough, 50 MW | APOS, U	Energy from solar thermal [kWh]	0.0085
Outputs:			
		Electricity mix M2040 [kWh]	1

LCI of global 2040 electricity mix was modeled in Ecoinvent according to the World Energy Outlook 2019 [2] projection for electricity mix in 2040.

Table S23. LCI of Global electricity mix. The grid mix considers the global share of different countries, according to the Ecoinvent database [1].

**Process:**	M2040 electricity mix		
	**Ecoinvent entry**	**Description**	**Amount**
Inputs:			
	Electricity, medium voltage {AU}| market for | APOS, U	Energy from coal [kWh]	0.010
	Electricity, medium voltage {NZ}| market for electricity, medium voltage | APOS, U	Energy from oil [kWh]	0.002
	Electricity, medium voltage {RAF}| market group for | APOS, U	Energy from natural gas [kWh]	0.032
	Electricity, medium voltage {RAS}| market group for | APOS, U	Energy from nuclear source [kWh]	0.461
	Electricity, medium voltage {RER}| market group for | APOS, U	Energy from hydropower [kWh]	0.173
	Electricity, medium voltage {RLA}| market group for | APOS, U	Energy from biomass [kWh]	0.062
	Electricity, medium voltage {RNA}| market group for | APOS, U	Energy from wind [kWh]	0.216
	Electricity, medium voltage {RoW}| market for | APOS, U	Energy from geothermal [kWh]	0.00008
	Electricity, medium voltage {RU}| market for | APOS, U	Energy from photovoltaic [kWh]	0.044
Outputs:			
		Electricity mix M2040 [kWh]	1

For combustion, the fuel consumption of 0.023 kg/t km was considered. [7,21] This value is within the range reported with fuel consumption at full load capacity for short-haul trucks between 0.018 and 0.024 kg diesel/t km. [22] In this case, our study, for the sake on simplicity will consider the same fuel consumption per t km, although this value has certain variations according to the type of fuel used.

Table S24. LCI of HDO100 combustion for freight road transport.

Process:	HDO100 combustion for freight road transport		
	**Ecoinvent entry**	**Description**	**Amount**
Inputs:			
	*HDO100 fuel	HDO100 fuel [kg]	0.023
Outputs:			
		Freight road transport [t km]	1
	Emissions to air	VOC, volatile organic compounds as C [kg]	2.17E-06
		Carbon monoxide, fossil [kg]	2.25E-05
		Nitrogen oxides [kg]	5.51E-05
		Particulates, < 10 um [kg]	1.24E-06
		Particulates, < 2.5 um [kg]	1.14E-06
		Sulfur oxides [kg]	0.00E+00
		Methane [kg]	2.34E-06
		Carbon dioxide, fossil [kg]	7.32E-02
		Dinitrogen monoxide [kg]	9.55E-08
		Carbon black [kg]	1.02E-07

LCI of HDO100 combustion was modeled in Ecoinvent based on the data provided by GREET® 2021 .Net software, emissions correspond to the combustion of a heavy-duty truck denominated “HD Truck: short haul” powered by HDO100 or renewable diesel.[23]

Table S25. LCI of diesel combustion for freight road transport.

Process:	DIESEL		
	**Ecoinvent entry**	**Description**	**Amount**
Inputs:			
	*Diesel fuel	Diesel fuel [kg]	0.023
Outputs:			
		Freight road transport [t km]	1
	Emissions to air	VOC, volatile organic compounds as C [kg]	2.17E-06
		Carbon monoxide, fossil [kg]	2.25E-05
		Nitrogen oxides [kg]	5.51E-05
		Particulates, < 10 um [kg]	1.24E-06
		Particulates, < 2.5 um [kg]	1.14E-06
		Sulfur oxides [kg]	5.21E-07
		Methane [kg]	2.34E-06
		Carbon dioxide, fossil [kg]	7.55E-02
		Dinitrogen monoxide [kg]	9.55E-08
		Carbon black [kg]	1.02E-07

LCI of diesel combustion was modeled in Ecoinvent based on the data provided by GREET® 2021 .Net software, emissions correspond to the combustion of a heavy-duty truck denominated “HD Truck: short haul” powered by low sulfur diesel.[23]

Table S26. LCI of biodiesel combustion for freight road transport.

Process:	BD20

	Ecoinvent entry	Description	Amount
Inputs:			
	*BD20 fuel	BD20 fuel [kg]	0.023
Outputs:			
		Freight road transport [tkm]	1
	Emissions to air	VOC, volatile organic compounds as C [kg]	2.17E-06
		Carbon monoxide, fossil [kg]	2.25E-05
		Nitrogen oxides [kg]	5.51E-05
		Particulates, < 10 um [kg]	1.24E-06
		Particulates, < 2.5 um [kg]	1.14E-06
		Sulfur oxides [kg]	4.17E-07
		Methane [kg]	2.34E-06
		Carbon dioxide, fossil [kg]	7.51E-02
		Dinitrogen monoxide [kg]	9.55E-08
		Carbon black [kg]	1.02E-07

LCI of BD20 combustion was modeled in Ecoinvent based on the data provided by GREET® 2021 .Net software, emissions correspond to the combustion of a heavy-duty truck denominated “HD Truck: short haul” powered by biodiesel 20% vol.[23]

3. Additional data

3.1. Overall level of transgression

The results shown in Figure 3 from the main manuscript are analyzed in greater detail for the different scenarios studied (Table S27 to Table S31). The planetary boundaries main contributions activities are detailed from Figure S1 to Figure S9, the resulting datasets generated during the current work are publicly available online at Cabrera-Jimenez R. et al.[24]

Table S27. Share of the global SOS, carbon footprint, human health impacts, and PB footprint for biodiesel production occupied by the global heavy-duty transport sector considering the current global electricity mix, 2040 sustainable electricity mix, and wind electricity.

Fuel	Scenario	aCO_2 ppm	EI Wm^{-2}	SOD DU	OA Ω_{arag}	P Tg P	N Tg N	LSC % LSC	FWU km^3	CBI % BII loss	CF kg CO_2 eq	HH DALY	PBF
BD20	$\mathrm{BLUE}_{C-CCS}^{\mathrm{M2020}}$	156%	148%	0%	9.99%	0.02%	1.06%	0.00%	1.26%	9.05%	4.3	752	5.06
BD20	$BLUE_{C-CCU}^{\mathrm{M2020}}$	141%	134%	0%	9.03%	0.02%	0.61%	0.00%	1.22%	8.09%	3.9	655	5.02
BD20	$GREY_{NoCCU}^{\mathrm{M2020}}$	173%	164%	0%	11.04%	0.02%	1.02%	0.00%	1.26%	10.02%	4.8	833	5.10
BD20	$\mathrm{GREEN}_{C-CCS}^{\mathrm{M2020}}$	110%	106%	0%	7.07%	0.02%	0.46%	0.00%	1.43%	6.62%	3.1	668	4.95
BD20	$\mathrm{GREEN}_{C-CCS}^{\mathrm{M2020}}$	118%	112%	0%	7.54%	0.02%	0.31%	0.00%	1.31%	6.85%	3.3	612	4.96
BD20	$\mathrm{YELLOW}_{NoCCU}^{\mathrm{M2020}}$	127%	121%	0%	8.11%	0.02%	0.42%	0.00%	1.43%	7.58%	3.6	750	4.99
BD20	$BLUE_{C-CCS}^{\mathrm{M2040}}$	147%	139%	0%	9.37%	0.02%	0.98%	0.00%	1.21%	8.75%	4.0	651	5.04
BD20	$BLUE_{C-CCU}^{\mathrm{M2040}}$	134%	126%	0%	8.55%	0.02%	0.55%	0.00%	1.18%	7.85%	3.6	576	5.00
BD20	$\mathrm{GREY}_{C-ACR}^{\mathrm{M2040}}$	160%	151%	0%	10.24%	0.02%	0.92%	0.00%	1.19%	9.62%	4.4	704	5.07
BD20	$GREEN_{C-CCS}^{\mathrm{M2040}}$	92%	87%	0%	5.89%	0.02%	0.31%	0.00%	1.34%	6.04%	2.6	476	4.90
BD20	$GREEN_{C-CCU}^{\mathrm{M2040}}$	106%	100%	0%	6.78%	0.02%	0.22%	0.00%	1.25%	6.48%	2.9	487	4.93
BD20	$\mathrm{YELLOW}_{NoCCU}^{\mathrm{M2040}}$	106%	100%	0%	6.76%	0.02%	0.25%	0.00%	1.33%	6.91%	2.9	529	4.93
BD20	$\mathrm{GREY}_{C-ACR}^{\mathrm{M2020}}$	159%	151%	0%	10.18%	0.02%	0.97%	0.00%	1.22%	9.13%	4.4	722	5.07
BD20	$GREY_{C-ACR}^{\mathrm{M2040}}$	152%	144%	0%	9.71%	0.02%	0.91%	0.00%	1.19%	8.90%	4.1	646	5.05
BD20	$YELLOW_{C-ACR}^{\mathrm{M2020}}$	113%	108%	0%	7.25%	0.02%	0.38%	0.00%	1.40%	6.70%	3.2	639	4.95
BD20	$YELLOW_{C-ACR}^{\mathrm{M2040}}$	97%	92%	0%	6.22%	0.02%	0.25%	0.00%	1.32%	6.19%	2.7	471	4.91
BD20	$\mathrm{BLUE}_{C-CCS}^{\mathrm{Wind}}$	145%	138%	0%	9.29%	0.02%	0.98%	0.00%	1.20%	8.33%	4.0	643	5.03
BD20	$BLUE_{C-CCU}^{\mathrm{Wind}}$	133%	126%	0%	8.49%	0.02%	0.55%	0.00%	1.17%	7.53%	3.6	570	5.00

BD20	$GREY^{Wind}_{NoCCU}$	159%	150%	0%	10.14%	0.02%	0.91%	0.00%	1.19%	9.09%	4.3	694	5.07
BD20	$GREEN^{Wind}_{C-CCS}$	90%	85%	0%	5.74%	0.02%	0.30%	0.00%	1.33%	5.25%	2.5	462	4.89
BD20	$GREEN^{Wind}_{C-CCU}$	105%	99%	0%	6.69%	0.02%	0.21%	0.00%	1.24%	5.97%	2.9	478	4.93
BD20	$YELLOW^{Wind}_{NoCCU}$	103%	97%	0%	6.59%	0.02%	0.24%	0.00%	1.31%	6.01%	2.8	513	4.92
BD20	$BLUE^{Wind}_{C-ACR}$	151%	143%	0%	9.65%	0.02%	0.91%	0.00%	1.18%	8.59%	4.1	640	5.05
BD20	$YELLOW^{Wind}_{C-ACR}$	95%	90%	0%	6.10%	0.02%	0.24%	0.00%	1.31%	5.51%	2.6	459	4.90

Rows in the table correspond to the scenario's biodiesel from microalgae. Acronyms for the scenario labels are as follows. BD20: Biodiesel 20% Vol; M2020: 2020 global electricity mix; M2040: Sustainable electricity mix for 2040; Wind: Electricity supplied from wind sources; DAC: CO_2 from Direct air capture; NGP: CO_2 from Natural gas power plant; CCU: Carbon capture and utilization of CO_2 from cogeneration by biomass combustion; CCS: Carbon capture and storage in a geological reservoir; C: cogeneration by biomass combustion; B: cogeneration by biogas combustion; Blue: NGP and either CCS or CCU is performed; Green: DAC and CCS or CCU is performed; Grey: NGP and CCS or CCU is not performed; Yellow: DAC and CCS or CCU is not performed; NoCCU: LEA cogeneration is not considered (aCO_2: atmospheric CO_2 concentration EI: energy imbalance at the top of the atmosphere; SOD: stratospheric ozone depletion; OA: ocean acidification; P: biogeochemical phosphorus flow- global; N: biogeochemical nitrogen flow-global; LSC: land-system change-global; FWU: freshwater use, global; CBI: biosphere integrity; CFP: Carbon footprint expressed in Gt CO_2eq; HH: Human health impacts expressed in DALYs; PBF: Planetary boundary footprint).

Table S28 Share of the global SOS, carbon footprint, Human health impacts, and PB footprint for biodiesel production occupied by global heavy-duty transport sector considering the current global electricity mix, 2040 sustainable electricity mix and wind electricity.

Fuel	Scenario	aCO_2 ppm	EI Wm^{-2}	SOD DU	OA Ω_{arag}	P Tg P	N Tg N	LSC % LSC	FWU km^3	CBI % BII loss	CF kg CO_2 eq	HH DALY	PBF
BD20	$BLUE^{M2020}_{B-CCS}$	160%	152%	0%	10.24%	0.02%	0.99%	0.00%	1.22%	9.35%	4.5	727	5.07
BD20	$BLUE^{M2020}_{B-CCU}$	152%	144%	0%	9.71%	0.02%	0.74%	0.00%	1.20%	8.82%	4.2	674	5.05
BD20	$GREY^{M2020}_{NoCCU}$	173%	164%	0%	11.04%	0.02%	1.02%	0.00%	1.26%	10.02%	4.8	833	5.10
BD20	$GREEN^{M2020}_{B-CCS}$	116%	110%	0%	7.40%	0.02%	0.39%	0.00%	1.40%	6.99%	3.3	648	4.96
BD20	$GREEN^{M2020}_{B-CCS}$	119%	113%	0%	7.59%	0.02%	0.31%	0.00%	1.33%	7.05%	3.4	613	4.97
BD20	$YELLOW^{M2020}_{NoCCU}$	127%	121%	0%	8.11%	0.02%	0.42%	0.00%	1.43%	7.58%	3.6	750	4.99
BD20	$BLUE^{M2040}_{B-CCS}$	155%	147%	0%	9.93%	0.02%	0.95%	0.00%	1.20%	9.20%	4.3	677	5.06
BD20	$BLUE^{M2040}_{B-CCU}$	148%	140%	0%	9.47%	0.02%	0.71%	0.00%	1.18%	8.70%	4.1	636	5.04
BD20	$GREY^{M2040}_{B-ACR}$	160%	151%	0%	10.24%	0.02%	0.92%	0.00%	1.19%	9.62%	4.4	704	5.07
BD20	$GREEN^{M2040}_{B-CCS}$	102%	97%	0%	6.54%	0.02%	0.28%	0.00%	1.33%	6.57%	2.9	507	4.92
BD20	$GREEN^{M2040}_{B-CCU}$	109%	103%	0%	6.95%	0.02%	0.23%	0.00%	1.28%	6.74%	3.1	509	4.94
BD20	$YELLOW^{M2040}_{NoCCU}$	106%	100%	0%	6.76%	0.02%	0.25%	0.00%	1.33%	6.91%	2.9	529	4.93
BD20	$GREY^{M2020}_{B-ACR}$	172%	163%	0%	10.99%	0.02%	0.94%	0.00%	1.20%	9.95%	4.8	743	5.10

BD20	$GREY^{M2040}_{B-ACR}$	168%	159%	0%	10.77%	0.02%	0.91%	0.00%	1.18%	9.84%	4.7	707	5.09
BD20	$YELLOW^{M2020}_{B-ACR}$	126%	120%	0%	8.06%	0.02%	0.34%	0.00%	1.38%	7.52%	3.6	660	4.99
BD20	$YELLOW^{M2040}_{B-ACR}$	114%	108%	0%	7.28%	0.02%	0.25%	0.00%	1.32%	7.14%	3.2	533	4.95
BD20	$\mathrm{BLUE}^{Wind}_{B-CCS}$	155%	147%	0%	9.89%	0.02%	0.95%	0.00%	1.19%	8.99%	4.3	674	5.06
BD20	$BLUE^{Wind}_{B-CCU}$	148%	140%	0%	9.44%	0.02%	0.71%	0.00%	1.18%	8.54%	4.1	633	5.04
BD20	$GREY^{Wind}_{NoCCU}$	159%	150%	0%	10.14%	0.02%	0.91%	0.00%	1.19%	9.09%	4.3	694	5.07
BD20	$\mathrm{GREEN}^{Wind}_{B-CCS}$	101%	95%	0%	6.43%	0.02%	0.28%	0.00%	1.32%	5.99%	2.9	497	4.92
BD20	$\mathrm{GREEN}^{Wind}_{B-CCU}$	107%	102%	0%	6.87%	0.02%	0.22%	0.00%	1.27%	6.32%	3.0	502	4.94
BD20	$\mathrm{YELLOW}^{Wind}_{NoCCU}$	103%	97%	0%	6.59%	0.02%	0.24%	0.00%	1.31%	6.01%	2.8	513	4.92
BD20	$BLUE^{Wind}_{B-ACR}$	168%	159%	0%	10.74%	0.02%	0.91%	0.00%	1.18%	9.70%	4.7	705	5.09
BD20	$YELLOW^{Wind}_{B-ACR}$	112%	107%	0%	7.19%	0.02%	0.24%	0.00%	1.31%	6.62%	3.2	523	4.95

Rows in the table correspond to the scenario's biodiesel from microalgae. Acronyms for the scenario labels are as follows. BD20: Biodiesel 20% Vol; M2020: 2020 global electricity mix; M2040: Sustainable electricity mix for 2040; Wind: Electricity supplied from wind sources; DAC: CO_2 from Direct air capture; NGP: CO_2 from Natural gas power plant; CCU: Carbon capture and utilization of CO_2 from cogeneration by biomass combustion; CCS: Carbon capture and storage in a geological reservoir; C: cogeneration by biomass combustion; B: cogeneration by biogas combustion; Blue: NGP and either CCS or CCU is performed; Green: DAC and CCS or CCU is performed; Grey: NGP and CCS or CCU is not performed; Yellow: DAC and CCS or CCU is not performed; NoCCU: LEA cogeneration is not considered (aCO_2: atmospheric CO_2 concentration EI: energy imbalance at the top of the atmosphere; SOD: stratospheric ozone depletion; OA: ocean acidification; P: biogeochemical phosphorus flow- global; N: biogeochemical nitrogen flow-global; LSC: land-system change-global; FWU: freshwater use, global; CBI: biosphere integrity; CFP: Carbon footprint expressed in Gt CO_2eq; HH: Human health impacts expressed in DALYs; PBF: Planetary boundary footprint).

Table S29. Share of the global SOS, carbon footprint, Human health impacts, and PB footprint for HTL production occupied by the global heavy-duty transport sector considering the current global electricity mix, 2040 sustainable electricity mix, and wind electricity.

		aCO_2	EI	SOD	OA	P	N	LSC	FWU	CBI	CF	HH	PBFs
**Fuel**	**Scenario**	**ppm**	**Wm^{-2}**	**DU**	**Ω_{arag}**	**Tg P**	**Tg N**	**% LSC**	**km^3**	**% BII loss**	**kg CO_2 eq**	**DALY**	
HTL	$\mathrm{BLUE}^{M2020}_{C-CCS}$	206%	196%	0%	13.15%	0.04%	2.24%	0.00%	0.54%	15.95%	5.8	1116	5.20
HTL	$BLUE^{M2020}_{C-CCU}$	179%	171%	0%	11.48%	0.04%	1.67%	0.00%	0.47%	14.27%	5.0	942	5.13
HTL	$GREY^{M2020}_{NoCCU}$	241%	229%	0%	15.39%	0.04%	2.08%	0.00%	0.48%	17.73%	6.7	1153	5.29
HTL	$\mathrm{GREEN}^{M2020}_{C-CCS}$	121%	117%	0%	7.73%	0.04%	0.98%	0.00%	0.93%	11.52%	3.6	992	4.98
HTL	$\mathrm{GREEN}^{M2020}_{C-CCS}$	109%	106%	0%	6.98%	0.04%	0.75%	0.00%	0.76%	10.58%	3.2	826	4.95
HTL	$\mathrm{YELLOW}^{M2020}_{NoCCU}$	145%	140%	0%	9.29%	0.04%	0.82%	0.00%	0.87%	12.71%	4.2	995	5.04
HTL	$BLUE^{M2040}_{C-CCS}$	178%	169%	0%	11.37%	0.04%	2.09%	0.00%	0.41%	15.08%	5.0	824	5.12
HTL	$BLUE^{M2040}_{C-CCU}$	156%	148%	0%	9.97%	0.04%	1.54%	0.00%	0.36%	13.53%	4.3	695	5.07

HTL	$\mathrm{GREY}^{\mathrm{M2040}}_{C-ACR}$	217%	206%	0%	13.88%	0.04%	1.95%	0.00%	0.37%	16.99%	6.0	906	5.22
HTL	$GREEN^{\mathrm{M2040}}_{C-CCS}$	74%	71%	0%	4.76%	0.04%	0.73%	0.00%	0.71%	10.07%	2.2	505	4.86
HTL	$GREEN^{\mathrm{M2040}}_{C-CCU}$	72%	68%	0%	4.59%	0.04%	0.54%	0.00%	0.58%	9.41%	2.1	435	4.85
HTL	$\mathrm{YELLOW}^{\mathrm{M2040}}_{NoCCU}$	103%	98%	0%	6.59%	0.04%	0.59%	0.00%	0.67%	11.39%	2.9	553	4.93
HTL	$\mathrm{BLUE}^{\mathrm{Wind}}_{C-CCS}$	174%	165%	0%	11.14%	0.04%	2.09%	0.00%	0.39%	13.84%	4.9	803	5.11
HTL	$BLUE^{\mathrm{Wind}}_{C-CCU}$	153%	145%	0%	9.77%	0.04%	1.54%	0.00%	0.34%	12.49%	4.2	677	5.06
HTL	$GREY^{Wind}_{NoCCU}$	214%	203%	0%	13.68%	0.04%	1.95%	0.00%	0.35%	15.94%	5.9	888	5.22
HTL	$\mathrm{GREEN}^{Wind}_{C-CCS}$	69%	65%	0%	4.38%	0.04%	0.73%	0.00%	0.67%	8.00%	2.0	470	4.84
HTL	$\mathrm{GREEN}^{Wind}_{C-CCS}$	67%	64%	0%	4.29%	0.04%	0.54%	0.00%	0.55%	7.75%	1.9	407	4.84
HTL	$\mathrm{YELLOW}^{Wind}_{NoCCU}$	98%	93%	0%	6.24%	0.04%	0.60%	0.00%	0.63%	9.51%	2.8	521	4.91

Rows in the table correspond to the scenario's HTL biofuel from microalgae. Acronyms for the scenario labels are as follows. BD20: Biodiesel 20% Vol; M2020: 2020 global electricity mix; M2040: Sustainable electricity mix for 2040; Wind: Electricity supplied from wind sources; DAC: CO_2 from Direct air capture; NGP: CO_2 from Natural gas power plant; CCU: Carbon capture and utilization of CO_2 from cogeneration by biomass combustion; CCS: Carbon capture and storage in a geological reservoir; C: cogeneration by biomass combustion; B: cogeneration by biogas combustion; Blue: NGP and either CCS or CCU is performed; Green: DAC and CCS or CCU is performed; Grey: NGP and CCS or CCU is not performed; Yellow: DAC and CCS or CCU is not performed; NoCCU: LEA cogeneration is not considered (aCO_2: atmospheric CO_2 concentration EI: energy imbalance at the top of the atmosphere; SOD: stratospheric ozone depletion; OA: ocean acidification; P: biogeochemical phosphorus flow- global; N: biogeochemical nitrogen flow-global; LSC: land-system change-global; FWU: freshwater use, global; CBI: biosphere integrity; CFP: Carbon footprint expressed in Gt CO_2eq; HH: Human health impacts expressed in DALYs; PBF: Planetary boundary footprint).

Table S30. Share of the global SOS, carbon footprint, human health impacts, and PB footprint for HDO100 production occupied by the global heavy-duty transport sector considering the current global electricity mix, 2040 sustainable electricity mix, and wind electricity.

		**aCO_2**	**EI**	**SOD**	**OA**	**P**	**N**	**LSC**	**FWU**	**CBI**	**CF**	**HH**	**PBFs**
**Fuel**	**Scenario**	**ppm**	**Wm^{-2}**	**DU**	**Ω_{arag}**	**Tg P**	**Tg N**	**% LSC**	**km^3**	**% BII loss**	**kg CO_2 eq**	**DALY**	
HDO100	$\mathrm{BLUE}^{\mathrm{M2020}}_{C-CCS}$	325%	310%	0%	20.76%	0.08%	4.54%	0.00%	5.46%	19.38%	9.2	1746	5.51
HDO100	$BLUE^{\mathrm{M2020}}_{C-CCU}$	255%	243%	0%	16.29%	0.08%	2.46%	0.00%	5.27%	14.91%	7.1	1295	5.32
HDO100	$GREY^{\mathrm{M2020}}_{NoCCU}$	400%	381%	0%	25.61%	0.08%	4.35%	0.00%	5.46%	23.85%	11.2	2125	5.70
HDO100	$\mathrm{GREEN}^{\mathrm{M2020}}_{C-CCS}$	112%	111%	0%	7.15%	0.08%	1.76%	0.00%	6.28%	8.07%	3.7	1357	4.96
HDO100	$\mathrm{GREEN}^{\mathrm{M2020}}_{C-CCS}$	146%	142%	0%	9.37%	0.08%	1.07%	0.00%	5.69%	9.15%	4.3	1095	5.05
HDO100	$\mathrm{YELLOW}^{\mathrm{M2020}}_{NoCCU}$	188%	182%	0%	12.01%	0.08%	1.58%	0.00%	6.29%	12.54%	5.6	1736	5.16
HDO100	$BLUE^{\mathrm{M2040}}_{C-CCS}$	279%	264%	0%	17.82%	0.08%	4.17%	0.00%	5.22%	17.93%	7.8	1266	5.39
HDO100	$BLUE^{\mathrm{M2040}}_{C-CCU}$	219%	207%	0%	13.99%	0.08%	2.17%	0.00%	5.09%	13.77%	6.0	919	5.23
HDO100	$\mathrm{GREY}^{\mathrm{M2040}}_{C-ACR}$	342%	322%	0%	21.86%	0.08%	3.89%	0.00%	5.17%	22.00%	9.4	1512	5.55

HDO100	$GREEN^{M2040}_{C-CCS}$	25%	25%	0%	1.62%	0.08%	1.07%	0.00%	5.85%	5.34%	1.1	454	4.74
HDO100	$GREEN^{M2040}_{C-CCU}$	90%	86%	0%	5.77%	0.08%	0.62%	0.00%	5.40%	7.38%	2.6	508	4.90
HDO100	$\mathrm{YELLOW}^{M2040}_{NoCCU}$	89%	83%	0%	5.66%	0.08%	0.78%	0.00%	5.79%	9.41%	2.7	700	4.90
HDO100	$\mathrm{GREY}^{M2020}_{C-ACR}$	338%	322%	0%	21.61%	0.08%	4.14%	0.00%	5.31%	19.74%	9.4	1608	5.54
HDO100	$GREY^{M2040}_{C-ACR}$	303%	287%	0%	19.38%	0.08%	3.86%	0.00%	5.13%	18.64%	8.4	1243	5.45
HDO100	$YELLOW^{M2020}_{C-ACR}$	125%	123%	0%	8.01%	0.08%	1.36%	0.00%	6.13%	8.43%	3.9	1219	4.99
HDO100	$YELLOW^{M2040}_{C-ACR}$	50%	48%	0%	3.18%	0.08%	0.76%	0.00%	5.75%	6.05%	1.6	431	4.80
HDO100	$\mathrm{BLUE}^{Wind}_{C-CCS}$	273%	259%	0%	17.46%	0.08%	4.15%	0.00%	5.19%	15.98%	7.7	1231	5.37
HDO100	$BLUE^{Wind}_{C-CCU}$	214%	203%	0%	13.70%	0.08%	2.16%	0.00%	5.07%	12.24%	5.9	892	5.22
HDO100	$GREY^{Wind}_{NoCCU}$	335%	315%	0%	21.40%	0.08%	3.86%	0.00%	5.13%	19.51%	9.2	1468	5.53
HDO100	$\mathrm{GREEN}^{Wind}_{C-CCS}$	15%	15%	0%	0.94%	0.08%	1.03%	0.00%	5.79%	1.66%	0.8	388	4.70
HDO100	$\mathrm{GREEN}^{Wind}_{C-CCU}$	83%	79%	0%	5.33%	0.08%	0.60%	0.00%	5.36%	4.98%	2.4	465	4.88
HDO100	$\mathrm{YELLOW}^{Wind}_{NoCCU}$	76%	71%	0%	4.88%	0.08%	0.74%	0.00%	5.72%	5.19%	2.3	625	4.86
HDO100	$BLUE^{Wind}_{C-ACR}$	299%	283%	0%	19.10%	0.08%	3.84%	0.00%	5.11%	17.15%	8.2	1217	5.44
HDO100	$YELLOW^{Wind}_{C-ACR}$	40%	39%	0%	2.58%	0.08%	0.72%	0.00%	5.70%	2.83%	1.3	374	4.77

Rows in the table correspond to the scenario's biofuel from microalgae. Acronyms for the scenario labels are as follows. BD20: Biodiesel 20% Vol; M2020: 2020 global electricity mix; M2040: Sustainable electricity mix for 2040; Wind: Electricity supplied from wind sources; DAC: CO_2 from Direct air capture; NGP: CO_2 from Natural gas power plant; CCU: Carbon capture and utilization of CO_2 from cogeneration by biomass combustion; CCS: Carbon capture and storage in a geological reservoir; C: cogeneration by biomass combustion; B: cogeneration by biogas combustion; Blue: NGP and either CCS or CCU is performed; Green: DAC and CCS or CCU is performed; Grey: NGP and CCS or CCU is not performed; Yellow: DAC and CCS or CCU is not performed; NoCCU: LEA cogeneration is not considered (aCO_2: atmospheric CO_2 concentration EI: energy imbalance at the top of the atmosphere; SOD: stratospheric ozone depletion; OA: ocean acidification; P: biogeochemical phosphorus flow- global; N: biogeochemical nitrogen flow-global; LSC: land-system change-global; FWU: freshwater use, global; CBI: biosphere integrity; CFP: Carbon footprint expressed in Gt CO_2eq; HH: Human health impacts expressed in DALYs; PBF: Planetary boundary footprint).

Table S31. Share of the global SOS, carbon footprint, human health impacts, and PB footprint for HDO100 production occupied by the global heavy-duty transport sector considering the current global electricity mix, 2040 sustainable electricity mix, and wind electricity.

Fuel	Scenario	aCO_2 ppm	EI Wm^{-2}	SOD DU	OA Ω_{arag}	P Tg P	N Tg N	LSC % LSC	FWU km^3	CBI % BII loss	CF kg CO_2 eq	HH DALY	PBFs
HDO100	$\mathrm{BLUE}^{M2020}_{B-CCS}$	342%	326%	0%	21.90%	0.08%	4.21%	0.00%	5.29%	20.75%	9.9	1631	5.55
HDO100	$BLUE^{M2020}_{B-CCU}$	304%	289%	0%	19.42%	0.08%	3.05%	0.00%	5.19%	18.28%	8.7	1383	5.45
HDO100	$GREY^{M2020}_{NoCCU}$	479%	454%	0%	30.61%	0.08%	4.35%	0.00%	5.46%	28.20%	13.3	2376	5.91
HDO100	$\mathrm{GREEN}^{M2020}_{B-CCS}$	136%	134%	0%	8.71%	0.08%	1.43%	0.00%	6.11%	9.80%	4.5	1263	5.02

HDO100	$\mathrm{GREEN}^{\mathrm{M2020}}_{B-CCS}$	150%	145%	0%	9.57%	0.08%	1.05%	0.00%	5.78%	10.08%	4.7	1100	5.06
HDO100	$\mathrm{YELLOW}^{\mathrm{M2020}}_{NoCCU}$	266%	255%	0%	17.00%	0.08%	1.58%	0.00%	6.29%	16.89%	7.7	1988	5.36
HDO100	$BLUE^{\mathrm{M2040}}_{B-CCS}$	319%	303%	0%	20.42%	0.08%	4.03%	0.00%	5.17%	20.02%	9.2	1390	5.49
HDO100	$BLUE^{\mathrm{M2040}}_{B-CCU}$	286%	271%	0%	18.28%	0.08%	2.91%	0.00%	5.10%	17.71%	8.2	1196	5.41
HDO100	$\mathrm{GREY}^{\mathrm{M2040}}_{B-ACR}$	420%	396%	0%	26.86%	0.08%	3.89%	0.00%	5.17%	26.35%	11.5	1764	5.75
HDO100	$GREEN^{\mathrm{M2040}}_{B-CCS}$	72%	70%	0%	4.64%	0.08%	0.92%	0.00%	5.79%	7.79%	2.6	599	4.86
HDO100	$GREEN^{\mathrm{M2040}}_{B-CCU}$	102%	98%	0%	6.56%	0.08%	0.67%	0.00%	5.54%	8.59%	3.3	608	4.93
HDO100	$\mathrm{YELLOW}^{\mathrm{M2040}}_{NoCCU}$	167%	157%	0%	10.66%	0.08%	0.78%	0.00%	5.79%	13.75%	4.8	952	5.10
HDO100	$\mathrm{GREY}^{\mathrm{M2020}}_{B-ACR}$	319%	304%	0%	20.39%	0.08%	3.99%	0.00%	5.20%	19.23%	9.2	1453	5.49
HDO100	$GREY^{\mathrm{M2040}}_{B-ACR}$	302%	287%	0%	19.31%	0.08%	3.85%	0.00%	5.12%	18.69%	8.7	1277	5.45
HDO100	$YELLOW^{\mathrm{M2020}}_{B-ACR}$	106%	105%	0%	6.79%	0.08%	1.21%	0.00%	6.03%	7.92%	3.6	1065	4.95
HDO100	$YELLOW^{\mathrm{M2040}}_{B-ACR}$	49%	48%	0%	3.11%	0.08%	0.75%	0.00%	5.74%	6.10%	1.9	465	4.79
HDO100	$\mathrm{BLUE}^{\mathrm{Wind}}_{B-CCS}$	316%	301%	0%	20.24%	0.08%	4.01%	0.00%	5.16%	19.04%	9.1	1373	5.02
HDO100	$BLUE^{\mathrm{Wind}}_{B-CCU}$	284%	269%	0%	18.14%	0.08%	2.90%	0.00%	5.09%	16.95%	8.1	1182	5.56
HDO100	$GREY^{\mathrm{Wind}}_{NoCCU}$	413%	389%	0%	26.40%	0.08%	3.86%	0.00%	5.13%	23.85%	11.3	1720	5.60
HDO100	$\mathrm{GREEN}^{Wind}_{B-CCS}$	65%	63%	0%	4.14%	0.08%	0.89%	0.00%	5.75%	5.08%	2.4	551	4.83
HDO100	$\mathrm{GREEN}^{\mathrm{Wind}}_{B-CCU}$	97%	93%	0%	6.19%	0.08%	0.65%	0.00%	5.51%	6.59%	3.1	573	4.92
HDO100	$\mathrm{YELLOW}^{Wind}_{NoCCU}$	154%	145%	0%	9.88%	0.08%	0.74%	0.00%	5.72%	9.53%	4.4	877	5.06
HDO100	$BLUE^{\mathrm{Wind}}_{B-ACR}$	300%	285%	0%	19.18%	0.08%	3.84%	0.00%	5.11%	17.98%	8.6	1264	5.15
HDO100	$YELLOW^{\mathrm{Wind}}_{B-ACR}$	42%	41%	0%	2.66%	0.08%	0.72%	0.00%	5.70%	3.66%	1.7	422	5.31

Rows in the table correspond to the scenario's biofuel from microalgae. Acronyms for the scenario labels are as follows. BD20: Biodiesel 20% Vol; M2020: 2020 global electricity mix; M2040: Sustainable electricity mix for 2040; Wind: Electricity supplied from wind sources; DAC: CO_2 from Direct air capture; NGP: CO_2 from Natural gas power plant; CCU: Carbon capture and utilization of CO_2 from cogeneration by biomass combustion; CCS: Carbon capture and storage in a geological reservoir; C: cogeneration by biomass combustion; B: cogeneration by biogas combustion; Blue: NGP and either CCS or CCU is performed; Green: DAC and CCS or CCU is performed; Grey: NGP and CCS or CCU is not performed; Yellow: DAC and CCS or CCU is not performed; NoCCU: LEA cogeneration is not considered (aCO_2: atmospheric CO_2 concentration EI: energy imbalance at the top of the atmosphere; SOD: stratospheric ozone depletion; OA: ocean acidification; P: biogeochemical phosphorus flow- global; N: biogeochemical nitrogen flow-global; LSC: land-system change-global; FWU: freshwater use, global; CBI: biosphere integrity; CFP: Carbon footprint expressed in Gt CO_2eq; HH: Human health impacts expressed in DALYs; PBF: Planetary boundary footprint).

3.2. Contributions to planetary boundaries

Figures S2 – S9 in this section we provide the breakdowns of every PB for the scenarios included in the main study with respect to the selected functional unit (FU), *i.e.*, the freight road transport (t km).

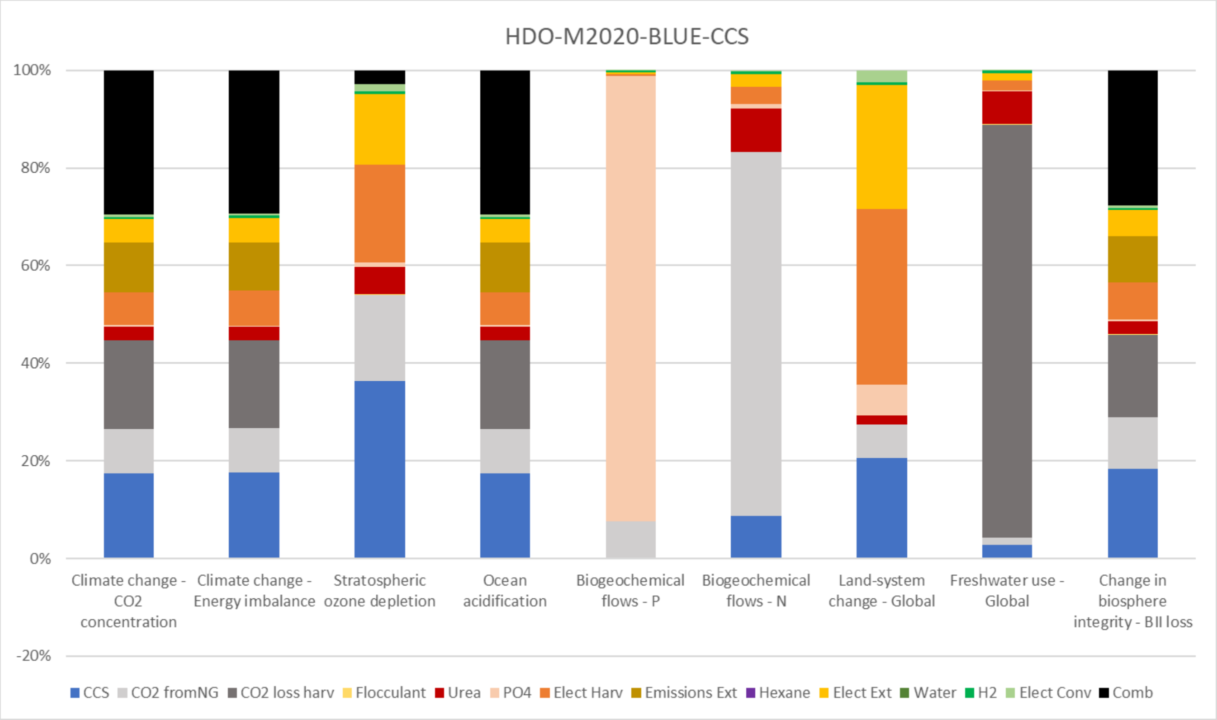

Figure S2. Breakdown of impact for planetary boundaries for $\text{HDO-BLUE}_{CCS}^{M2020}$ (FU=1 t km).

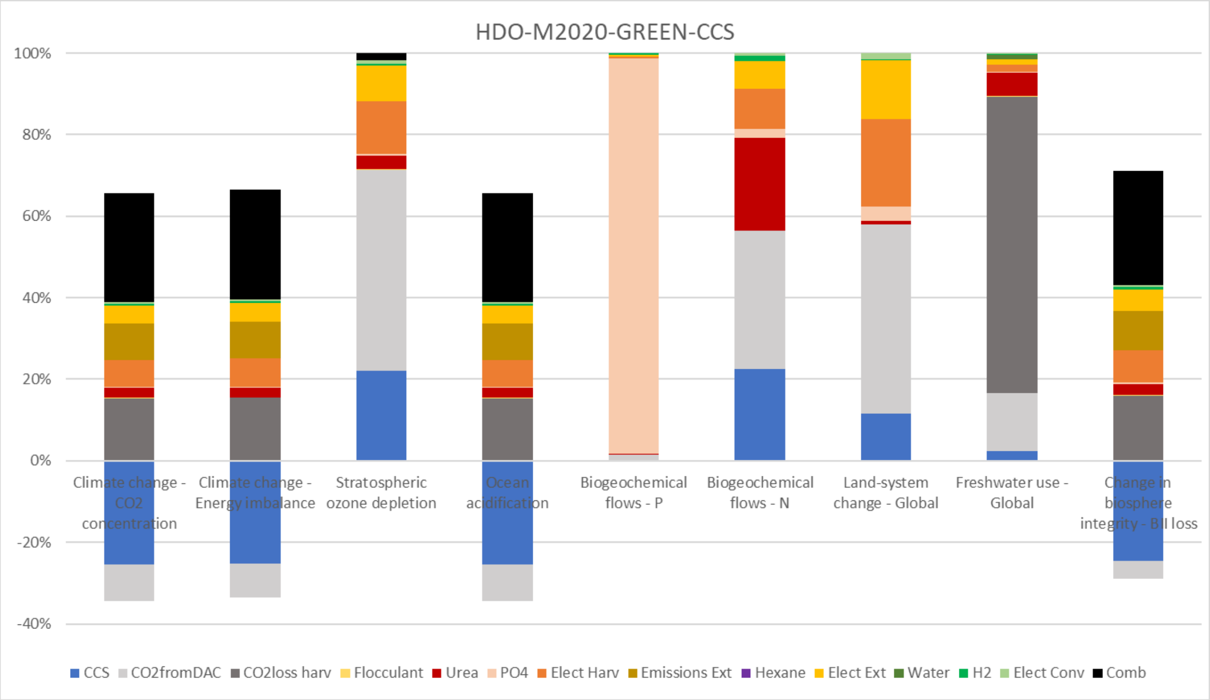

Figure S3. Breakdown of impact for planetary boundaries for $\text{HDO} - \text{GREEN}_{CCS}^{M2020}$ (FU=1 t km).

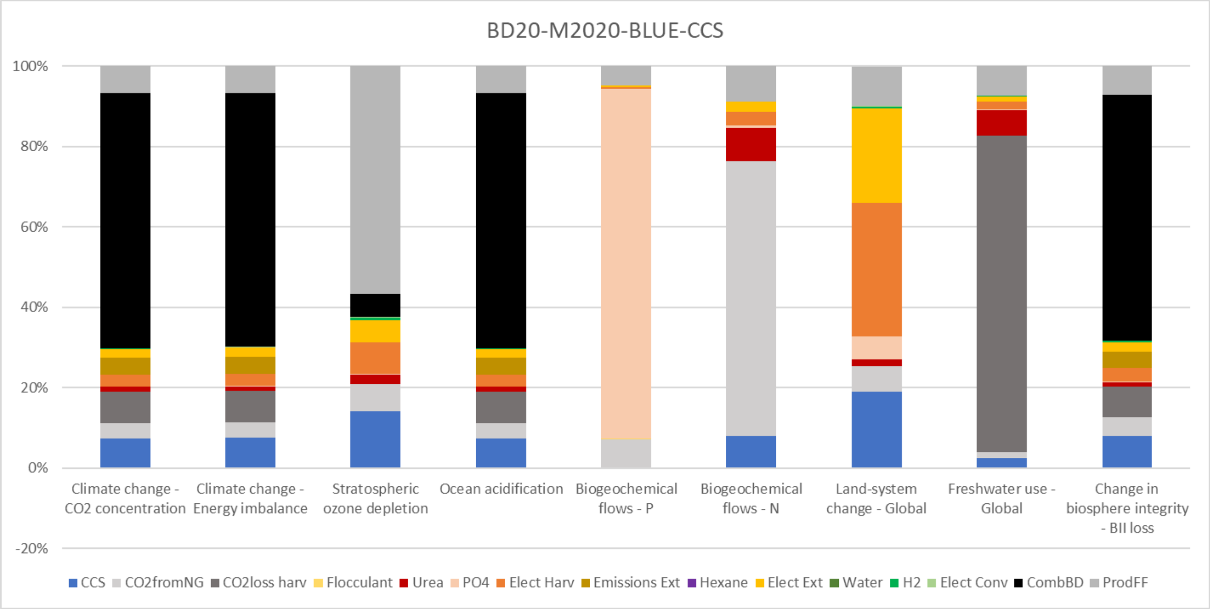

Figure S4. Breakdown of impact for planetary boundaries for $\mathrm{BD20} - \mathrm{BLUE}_{CCS}^{\mathrm{M2020}}$(FU=1 t km), the biodiesel fuel considered a blend with diesel 20% vol. BD.

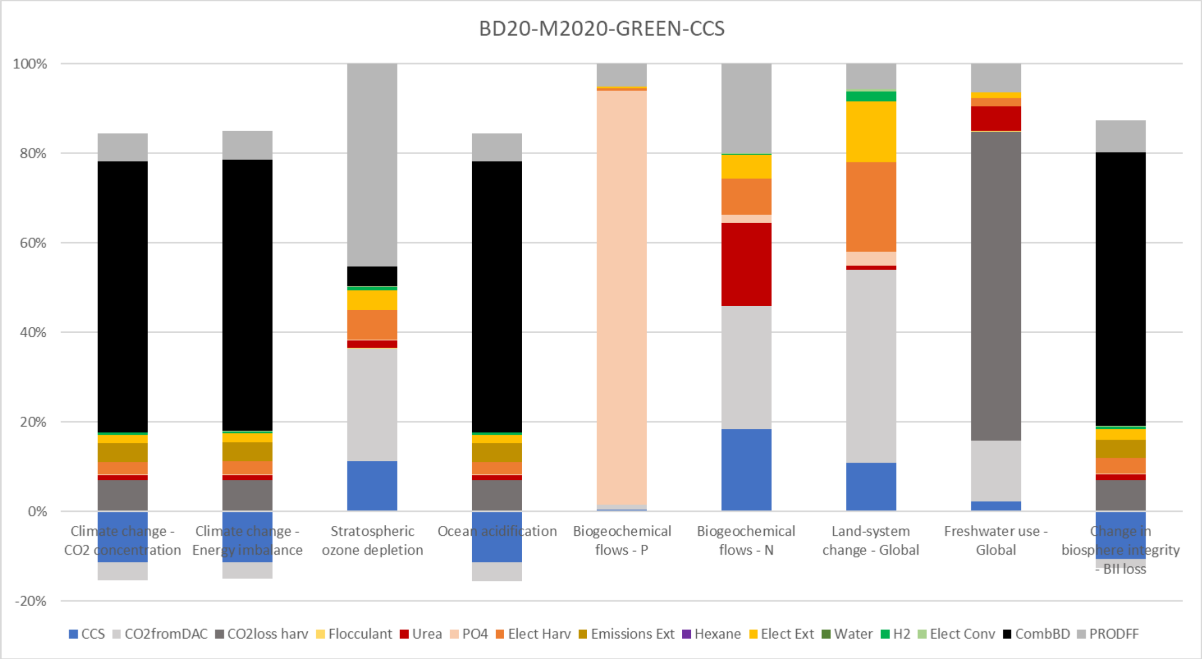

Figure S5. Breakdown of impact for planetary boundaries for $\mathrm{BD20} - \mathrm{GREEN}_{CCS}^{\mathrm{M2020}}$ (FU=1 t km), the biodiesel fuel considered a blend with diesel 20% vol. BD.

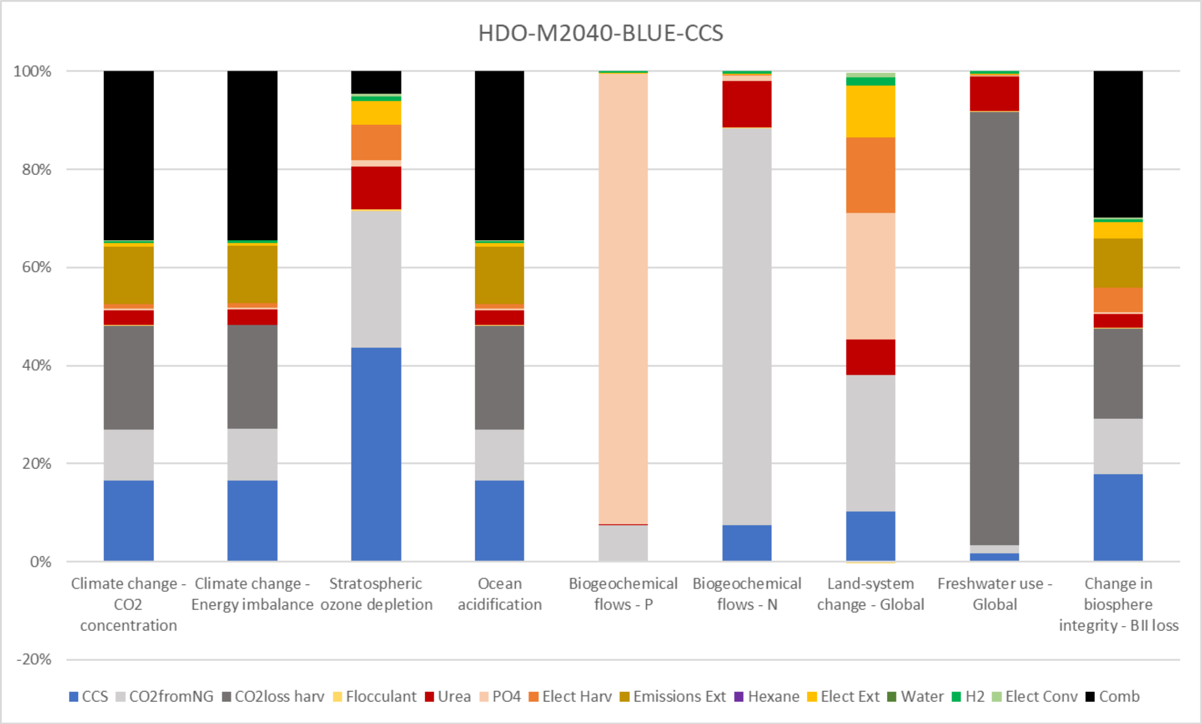

Figure S6. Breakdown of impact for planetary boundaries for $\mathrm{HDO}-\mathrm{BLUE}_{CCS}^{\mathrm{M2040}}$ (FU=1 t km)

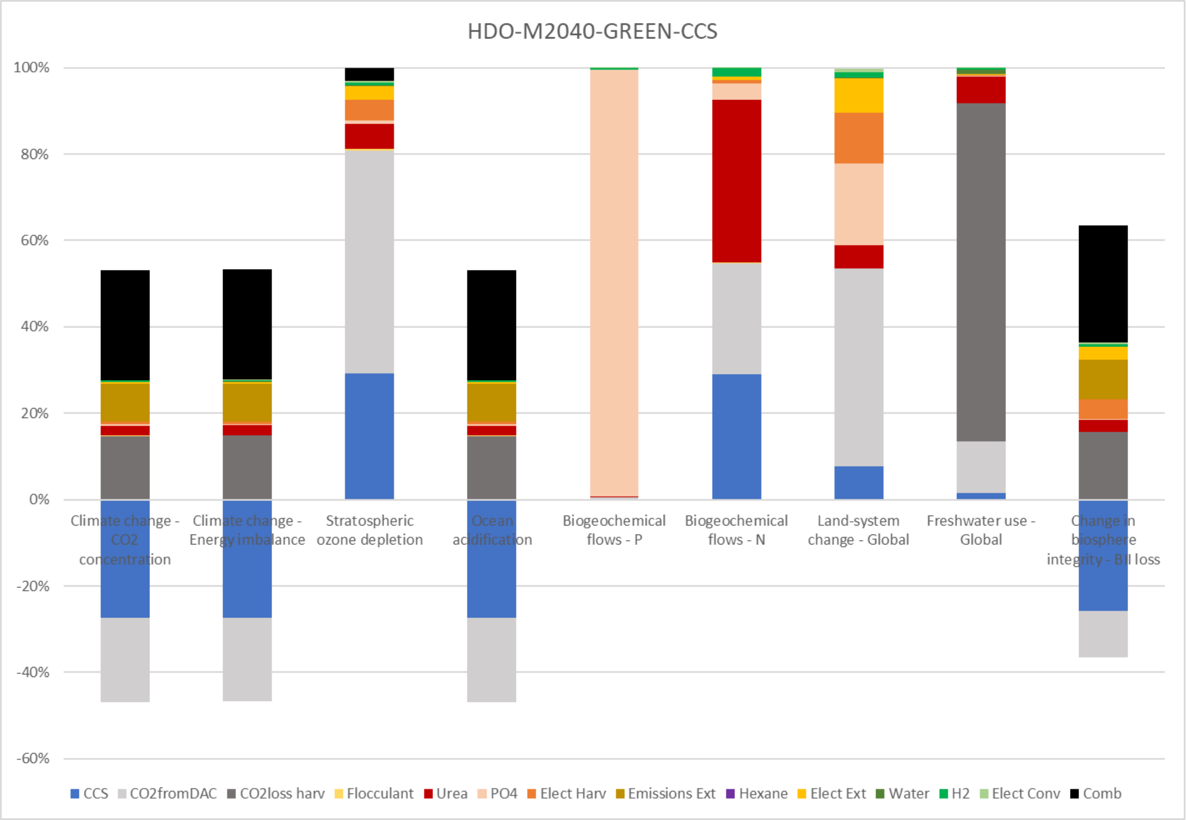

Figure S7. Breakdown of impact for planetary boundaries for $\mathrm{HDO}-\mathrm{GREEN}_{CCS}^{\mathrm{M2040}}$ (FU=1 t km).

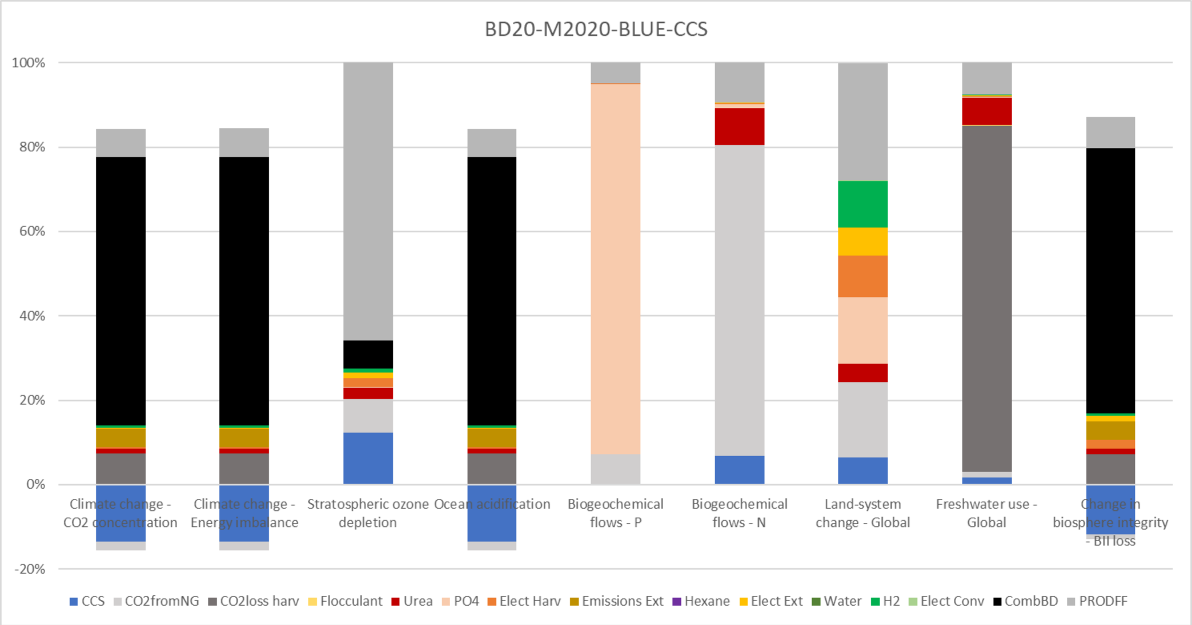

Figure S8. Breakdown of impact for planetary boundaries for $\mathrm{BD20-BLUE}_{CCS}^{\mathrm{M2020}}$ (FU=1 t km), the biodiesel fuel considered a blend with diesel 20% vol. BD.

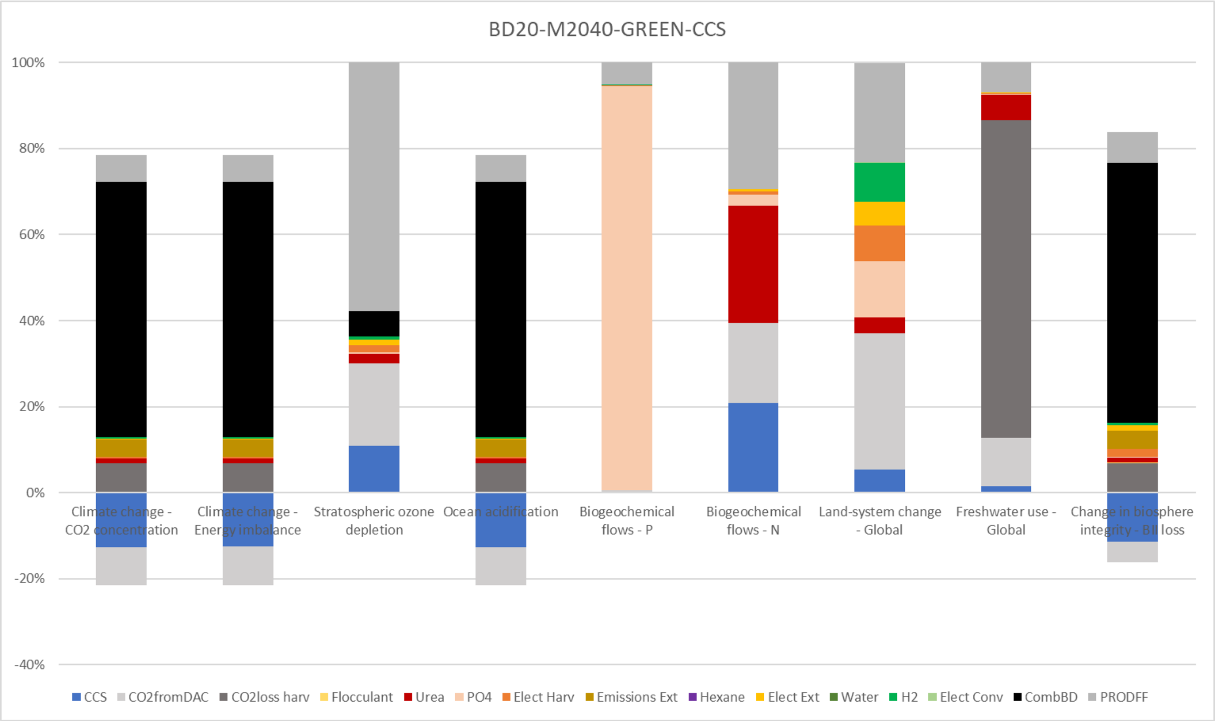

Figure S9. Breakdown of impact for planetary boundaries for $\mathrm{BD20-GREEN}_{CCS}^{\mathrm{M2040}}$ (FU=1 t km), the biodiesel fuel considered a blend with diesel 20% vol. BD.

Table S32. Technology readiness level of the main microalgae harvesting and extraction technologies

Stage	Technology	TRL	Reference
Cultivation			
	Open ponds	9	[7,25]
	Hanging bags	7	[7]
	Glass helical	7	[7]
	Horizontal Tubular	7	[7]
Harvesting			
	Floculation	9	[7]
	Centrigugation	9	[7,26]
	Acoustic harvesting	6	[27]
Extraction			
	Dry extraction (Hexane)	9	[28]
	Wet extraction (Hexane, methanol)	9	[28]
	Sonification	4	[29]
	Supercritical CO_2	3	[29]

References

(1) Wernet, G.; Bauer, C.; Steubing, B.; Reinhard, J.; Moreno-Ruiz, E.; Weidema, B. The Ecoinvent Database Version 3 (Part I): Overview and Methodology. *Int J Life Cycle Assess* **2016**, *21* (9), 1218–1230. https://doi.org/10.1007/S11367-016-1087-8.

(2) Energy Agency, I. *World Energy Outlook 2019*. www.iea.org/weo (accessed 2022-06-21).

(3) Petrakopoulou, F.; Iribarren, D.; Dufour, J. Life-Cycle Performance of Natural Gas Power Plants with Pre-Combustion CO2 Capture. *Greenhouse Gases: Science and Technology* **2015**, *5* (3), 268–276. https://doi.org/10.1002/GHG.1457.

(4) Keith, D. W.; Holmes, G.; St. Angelo, D.; Heidel, K. A Process for Capturing CO2 from the Atmosphere. *Joule* **2018**, *2* (8), 1573–1594. https://doi.org/10.1016/J.JOULE.2018.05.006.

(5) Bello, S.; Galán-Martín, Á.; Feijoo, G.; Moreira, M. T.; Guillén-Gosálbez, G. BECCS Based on Bioethanol from Wood Residues: Potential towards a Carbon-Negative Transport and Side-Effects. *Appl Energy* **2020**, *279*, 115884. https://doi.org/10.1016/J.APENERGY.2020.115884.

(6) Bau, D.; Und Geomatik, U.; Wildbolz, C. *Life Cycle Assessment of Selected Technologies for CO2 Transport and Sequestration Swiss Federal Institute of Technology Zurich*; 2007. http://www.ieagreen.org.uk/march81.htmAccessDate:18.Juni07.WeyburnProject. (accessed 2022-06-15).

(7) *Argonne GREET Model*. https://greet.es.anl.gov/ (accessed 2022-10-28).

(8) Zhu, Y.; Jones, S. B.; Schmidt, A. J.; Job, H. M.; Billing, J. M.; Collett, J. R.; Pomraning, K. R.; Fox, S. P.; Hart, T. R.; Edmundson, S. J.; Thorson, M. R.; Meyer, P. A.; Snowden-Swan,

L. J.; Anderson, D. B. Microalgae Conversion to Biofuels and Biochemical via Sequential Hydrothermal Liquefaction (SEQHTL) and Bioprocessing: 2020 State of Technology. **2021**. https://doi.org/10.2172/1784347.

(9) Cabrera-Jiménez, R.; Mateo-Sanz, J. M.; Gavaldà, J.; Jiménez, L.; Pozo, C. Comparing Biofuels through the Lens of Sustainability: A Data Envelopment Analysis Approach. *Appl Energy* **2022**, *307*, 118201. https://doi.org/10.1016/J.APENERGY.2021.118201.

(10) Davis, R.; Markham, J.; Kinchin, C.; Zhu, Y.; Jones, S.; Han, J.; Canter, C.; Li, Q. *Algae Harmonization Study: Evaluating the Potential for Future Algal Biofuel Costs, Sustainability, and Resource Assessment from Harmonized Modeling*; 2021. https://www.nrel.gov/docs/fy18osti/70715.pdf. (accessed 2023-03-28).

(11) Davis, R.; Wiatrowski, M.; Kinchin, C.; Humbird, D. *Conceptual Basis and Techno-Economic Modeling for Integrated Algal Biorefinery Conversion of Microalgae to Fuels and Products*; 2020. https://www.nrel.gov/docs/fy20osti/75168.pdf. (accessed 2023-03-29).

(12) Zaimes, G. G.; Khanna, V. Microalgal Biomass Production Pathways: Evaluation of Life Cycle Environmental Impacts. *Biotechnol Biofuels* **2013**, *6* (1), 1–11. https://doi.org/10.1186/1754-6834-6-88/TABLES/2.

(13) Moody, J. W.; McGinty, C. M.; Quinn, J. C. Global Evaluation of Biofuel Potential from Microalgae. *Proc Natl Acad Sci U S A* **2014**, *111* (23), 8691–8696. https://doi.org/10.1073/PNAS.1321652111/-/DCSUPPLEMENTAL/PNAS.1321652111.SAPP.PDF.

(14) Argonne National Laboratory. *GREET Excel Model Platform*. Energy Systems and Infrastructure Analysis. https://greet.es.anl.gov/greet_excel_model.models (accessed 2023-04-10).

(15) Frank, E. D.; Han, J.; Palou-Rivera. I; Elgowainy, A.; Wang, M. Q. *Life-Cycle Analysis of Algal Lipid Fuels with the GREET Model*; Oak Ridge, 2011. https://greet.es.anl.gov/files/algal-lipid-fuels.

(16) Davis, R.; Markham, J.; Kinchin, C.; Zhu, Y.; Jones, S.; Han, J.; Canter, C.; Li, Q. *2017 Algae Harmonization Study: Evaluating the Potential for Future Algal Biofuel Costs, Sustainability, and Resource Assessment from Harmonized Modeling Contributing Authors Algae Farm TEA: CAP Conversion TEA: HTL Conversion TEA: System LCA*; 2021. https://www.nrel.gov/docs/fy18osti/70715.pdf. (accessed 2022-06-15).

(17) Ioannou, I.; D'Angelo, S. C.; Martín, A. J.; Pérez-Ramírez, J.; Guillén-Gosálbez, G. Hybridization of Fossil- and CO2-Based Routes for Ethylene Production Using Renewable Energy. *ChemSusChem* **2020**, *13* (23), 6370–6380. https://doi.org/10.1002/CSSC.202001312.

(18) Iribarren, D.; Petrakopoulou, F.; Dufour, J. Environmental and Thermodynamic Evaluation of CO2 Capture, Transport and Storage with and without Enhanced Resource Recovery. *Energy* **2013**, *50* (1), 477–485. https://doi.org/10.1016/J.ENERGY.2012.12.021.

(19) González-Garay, A.; Frei, M. S.; Al-Qahtani, A.; Mondelli, C.; Guillén-Gosálbez, G.; Pérez-Ramírez, J. Plant-to-Planet Analysis of CO 2 -Based Methanol Processes. *Energy Environ Sci* **2019**, *12* (12), 3425–3436. https://doi.org/10.1039/C9EE01673B.

(20) Albrecht, K. O.; Zhu, Y.; Schmidt, A. J.; Billing, J. M.; Hart, T. R.; Jones, S. B.; Maupin, G.; Hallen, R.; Ahrens, T.; Anderson, D. Impact of Heterotrophically Stressed Algae for Biofuel Production via Hydrothermal Liquefaction and Catalytic Hydrotreating in Continuous-Flow Reactors. *Algal Res* **2016**, *14*, 17–27. https://doi.org/10.1016/J.ALGAL.2015.12.008.

(21) Earl, T.; Mathieu, L.; Cornelis, S.; Kenny, S.; Ambel, C. C.; Nix, J. Analysis of Long Haul Battery Electric Trucks in EU. *8th Commercial Vehicle Workshop* **2018**, 17–18.

(22) Mårtensson, L. *Emissions from Volvo's trucks*. www.volvotrucks.com. (accessed 2022-10-28).

(23) Argonne National Laboratory. *GREET life cycle analysis tool*. Energy Systems and Infrastructure Analysis. https://greet.es.anl.gov/index.php?content=greetdotnet (accessed 2023-04-10).

(24) Cabrera-Jimenez, R.; Tulus, V.; Gavalda, J.; Jiménez, L.; Guillén-Gosálbez, G.; Pozo, C. Dataset: The Implications of Microalgae Biofuel Production for the Heavy-Duty Transport Sector under Planetary Boundary Perspective. 2023. https://doi.org/10.5281/zenodo.7850070.

(25) Harvey J, P. *The Microalgae Biorefinery*; 2017. https://cordis.europa.eu/docs/results/613/613870/final1-dfactory-final-final-all-report.pdf (accessed 2022-09-14).

(26) Wicker, R. J.; Kumar, G.; Khan, E.; Bhatnagar, A. Emergent Green Technologies for Cost-Effective Valorization of Microalgal Biomass to Renewable Fuel Products under a Biorefinery Scheme. *Chemical Engineering Journal* **2021**, *415*, 128932. https://doi.org/10.1016/J.CEJ.2021.128932.

(27) Shi, R.; Handler, R. M.; Shonnard, D. R. Life Cycle Assessment of Novel Technologies for Algae Harvesting and Oil Extraction in the Renewable Diesel Pathway. *Algal Res* **2019**, *37*, 248–259. https://doi.org/10.1016/J.ALGAL.2018.12.005.

(28) Lardon, L.; Hélias, A.; Sialve, B.; Steyer, J. P.; Bernard, O. Life-Cycle Assessment of Biodiesel Production from Microalgae. *Environ Sci Technol* **2009**, *43* (17), 6475–6481. https://doi.org/10.1021/ES900705J/SUPPL_FILE/ES900705J_SI_001.PDF.

(29) Brentner, L. B.; Eckelman, M. J.; Zimmerman, J. B. Combinatorial Life Cycle Assessment to Inform Process Design of Industrial Production of Algal Biodiesel. *Environ Sci Technol* **2011**, *45* (16), 7060–7067. https://doi.org/10.1021/ES2006995/SUPPL_FILE/ES2006995_SI_001.PDF.
